# Safety and Immunogenicity of Intranasal Razi Cov Pars as a COVID-19 Booster Vaccine in Adults: Promising Results from a Groundbreaking Clinical Trial

**DOI:** 10.3390/vaccines12111255

**Published:** 2024-11-05

**Authors:** Mohammad Hossein Fallah Mehrabadi, Monireh Hajimoradi, Ali Es-haghi, Saeed Kalantari, Mojtaba Noofeli, Ali Rezaei Mokarram, Seyed Hossein Razzaz, Maryam Taghdiri, Ladan Mokhberalsafa, Fariba Sadeghi, Vahideh Mohseni, Safdar Masoumi, Rezvan Golmoradi-Zadeh, Mohammad Hasan Rabiee, Masoud Solaymani-Dodaran, Seyed Reza Banihashemi

**Affiliations:** 1 Department of Epidemiology, Razi Vaccine and Serum Research Institute, Agricultural Research, Education and Extension Organization (AREEO), P.O. Box 31975/148, Karaj, Iranrabiee.hasan@yahoo.com (M.H.R.); 2 Department of Immunology, Razi Vaccine and Serum Research Institute, Agricultural Research, Education and Extension Organization (AREEO), P.O. Box 31975/148, Karaj, Iran; hajimoradimonireh@gmail.com (M.H.);; 3 Department of Physico Chemistry, Razi Vaccine and Serum Research Institute, Agricultural Research, Education and Extension Organization (AREEO), P.O. Box 31975/148, Karaj, Iran; 4 Departments of Infectious Diseases and Tropical Medicine, Iran University of Medical Sciences, P.O. Box 354-14665, Tehran, Iran; 5 Department of Recombinant Antigens Research, Razi Vaccine and Serum Research Institute, Agricultural Research, Education and Extension Organization (AREEO), P.O. Box 31975/148, Karaj, Iran; 6 Department of Adjuvant and formulation Research, Razi Vaccine and Serum Research Institute, Agricultural Research, Education and Extension Organization (AREEO), P.O. Box 31975/148, Karaj, Iran; 7 Clinical Trial and Ethics Group, Department of QA, Razi Vaccine and Serum Research Institute, Agricultural Research, Education and Extension Organization (AREEO), P.O. Box 31975/148, Karaj, Iran; 8 Department of QC, Razi Vaccine and Serum Research Institute, Agricultural Research, Education and Extension Organization (AREEO), P.O. Box 31975/148, Karaj, Iran; 9 School of Public Health, Iran University of Medical Sciences, P.O. Box 354-14665, Tehran, Iran; 10 Department of Biostatistics, Tarbiat Modares University, P.O. Box 14115-111, Tehran, Iran; 11 Department of Microbiology, School of Medicine, Iran University of Medical Sciences, P.O. Box 354-14665, Tehran, Iran; 12 Department of Epidemiology, Iran University of Medical Sciences, P.O. Box 354-14665, Tehran, Iran

**Keywords:** intranasal recombinant protein subunit vaccine, mucosal immunity, COVID-19, Razi Cov Pars, booster, clinical trial

## Abstract

Protective antibodies in the upper respiratory tract prevent the spread of COVID-19 in the community. Intranasal vaccines could raise the specific secretory IgA and IgG levels. This is a single-center, randomized, double-blind, placebo-controlled clinical trial to evaluate the safety and immunogenicity of Razi Cov Pars (RCP) intranasal recombinant protein subunit COVID-19 vaccine as a booster in adults. We compared specific IgG and IgA levels in the intranasal RCP group (*n* = 97) versus placebo (*n* = 96) in serum, saliva, and nasal mucosal secretions on days 0 and 14 and reported their Geometric Mean Ratios (GMR) and 95% confidence intervals (CI). We showed significant increases in IgA and IgG anti-RBD in the nasal mucosa in the RCP group, but their increase was not detectable in the serum and saliva. Anti-spike IgA in the nasal mucosa also increased in the RCP group compared to the placebo. This increase against the COVID-19 variant Omicron was also similar to that of the Wuhan. We detected no serious adverse reactions or anaphylaxis and all adverse events resolved completely during the follow-up period and were similar in both groups. Intranasal RCP is safe, stimulates the respiratory mucosal immunity, and could be a booster on various COVID-19 vaccines and be effective against new virus variants.

## 1. Introduction

The world witnessed a significant number of deaths due to the COVID-19 pandemic [1], and the implemented control measures had a profound impact on the economy [2]. Vaccination has proved to be an effective tool in combating the virus, protecting millions of individuals, and mitigating the severity of the pandemic [3]. However, despite the widespread use of various vaccination platforms, the virus continued to spread due to the limited ability of existing vaccines to stimulate the immune response of the upper respiratory tract [4] and the emergence of new variants. 

Intranasal COVID-19 vaccines offer a promising solution to overcome the limitations of current vaccines. Intranasal vaccines mimic the virus’s natural entry pathway into the body [5]. This unique delivery method can provide enhanced protection against infection compared to injected vaccines [6]. Furthermore, intranasal vaccines can be more effective in preventing virus transmission. By generating a robust immune response in the upper respiratory tract, where the virus primarily replicates, these vaccines can reduce the number of infections and slow down the pandemic’s spread [7].

Several companies, including Razi Institute in Iran, are actively developing intranasal COVID-19 vaccines. RCP (Razi Cov Pars), a leading intranasal vaccine based on Nano systemic adjuvanted recombinant protein technology [8], is administered via both intramuscular and intranasal routes. Derived from the Wuhan variant of SARS-CoV-2, this vaccine has exhibited a favorable safety and efficacy profile in multiple clinical trials [9,10,11]. It received approval for use in Iran in February 2021 (comprising two intramuscular and one intranasal dose) and has been made accessible to the general population.

Building on promising results from prior studies [10], this research aims to provide further evidence regarding the safety and efficacy of intranasal RCP administration for improving the mucosal immune response of the upper respiratory tract. The study seeks to establish RCP’s potential as a booster across different vaccine platforms by comparing specific IgG and IgA antibody responses in serum, saliva, and nasal mucosal secretions of participants who received intranasal RCP versus an intranasal placebo in a randomized, double-blind, placebo-controlled clinical trial.

## 2. Materials and Methods 

### 2.1. Trial Design

This is a single-centered, randomized, double-blind, placebo-controlled clinical trial exploring the safety and immunogenicity of intranasal RCP, a recombinant protein subunit COVID-19 vaccine, as a booster dose in the adult population. Eligible participants who had received their last COVID-19 vaccinations five months or more before entering the current study received an intranasal booster dose of RCP or a placebo via a nasal delivery device. The study setting was a health clinic located in the RAZI Vaccine and Serum Research Institute Health Clinic in Karaj, Iran. We received the ethical approval of this study from the National Research Ethics Committee (NREC) (Ref: IR.NREC.1401.004) and registered with the Iranian Registry of Clinical Trials (IRCT) (Ref: IRCT20201214049709N6).

### 2.2. Participants

Participants were adult Iranian men and non-pregnant women aged 18 years or older who had been vaccinated at least five months before this study with one of the locally approved COVID-19 vaccines and did not have a history of COVID-19 infection in the last six months. 

Women of reproductive age (18 to 49 years) should have agreed to use at least one reliable contraceptive method (condom, oral contraceptive pills, intrauterine contraceptive device, IUD, Norplant capsule) and have a negative pregnancy test (baby check) on the day of vaccination. 

The main exclusion criteria were: close contact with a confirmed COVID-19 case within the last two weeks; history of allergic reactions to any drug or vaccine; breastfeeding; having any acute or chronic illness requiring continuous ongoing medical or surgical care, or unstable diseases within the last six months; history of severe cardiovascular diseases; uncontrolled serious psychiatric illnesses; blood transfusion or receiving any blood products or immunoglobulins within the last three months; having immunocompromising illnesses; history of long-term use of immunosuppressive drugs or systemic corticosteroids equivalent to 10 mg or more daily prednisolone for more than 14 consecutive days within the last four months; recent diagnosis or treatment of cancers; current substance or alcohol abuse; and splenectomy for any reason.

### 2.3. Randomization and Masking

We used block randomization stratified by the type of the last vaccine received (seven randomization chains for seven vaccine types). The block sizes were four and six, and their sequences were random. Unique 8-digit concealment codes were used to mask the type of intervention allocated to each participant (intranasal RCP or a placebo). The codes were delivered via a web-based study software that we used to manage every aspect of the study, from the first participant’s online enrollment to the end of the last follow-up call. When a participant reached the vaccine delivery stage, the type of the allocated intervention was displayed on a computer screen for a short while for the trial nurse. It was hidden again immediately after the confirmation of the intranasal delivery. All other study staff were kept blind to the intervention type. We also masked the identity of the participants in all specimens for the immunology lab officers. Every specimen sent to the immunology lab was uniquely coded to remove the possibility of identification of emerging patterns in the lab results for each participant across different time points and specimen types. 

### 2.4. Procedure

Participants, were enrolled via a website, passed an online screening, and were invited to the trial unit. They were then asked to sign an informed consent form and underwent further on-site screening. Eligible participants were randomly assigned to the intervention or control groups. The intervention group received one dose of 10 μg/200 μL intranasal RCP, a recombinant protein subunit COVID-19 vaccine, via a nasal delivery device. The control group received a placebo that was an adjuvant-only preparation and delivered intra-nasally in the same manner as the vaccine. 

All participants were requested to keep their heads up for a minute and avoid eating and drinking for 30 min. They were closely observed for half an hour immediately after receiving the intervention for any acute anaphylactic reaction, and their vital signs were monitored.

We used a mobile application integrated into the study software to collect the follow-up information. Systemic adverse reactions such as nausea, vomiting, headache, myalgia, and fatigue were recorded daily for seven days. We followed the participants for a month through weekly telephone calls, and all medically attended adverse events were recorded using the mobile application. 

We took blood, saliva, and nasal mucosal secretion samples before administration of the intervention and 14 days later. We measured IgA and IgG antibody responses against specific S and RBD (Receptor Binding Domain) antigens using house ELISA kits and specific COVID-19 antigens (Native Antigen, Oxford, UK). We tested six dilutions (0.1, 0.01, 0.001, 0.0001, 0.00001, and 0.000001) for each specimen and calculated the area under the curve (AUC). Resistance against changing variants was explored using S-Trimer antigenic components (Native Antigen, Oxford, UK) of the Wuhan and Omicron SARS-CoV-2 variants. (Please see the Supplementary Materials for more details about immunogenicity assessment methods).

### 2.5. Outcomes 

The immunogenicity outcomes were the specific antibody responses in serum, saliva, and nasal mucosal secretions two weeks after the intranasal dose. Initially, only saliva sampling was planned to assess the upper respiratory tract mucosal immune response. However, due to challenges in obtaining real saliva samples and the risk of microbial contaminations and potential interference of buccal enzymes in test results [12,13,14], nasal swab samples were included to collect nasal mucosal secretions. Specific IgG and IgA antibody responses were assessed against the COVID-19 spike and RBD. Additionally, we compared specific IgA antibody responses against spike antigens from Wuhan and Omicron variants of COVID-19 in the nasal mucosal secretion. 

The safety outcomes were abnormal vital signs and anaphylactic reactions immediately after vaccination (within the first half an hour), systemic solicited adverse reactions within the first week post-vaccination, and serious adverse events up to one month after the vaccination. We collected information on all medically attended adverse events (MAAEs) for all study subjects in the month following the vaccination. We categorized solicited local and systemic adverse reactions using FDA toxicity grading scales [15]. We assessed the detected adverse events during the one-month follow-up period regarding causality [16].

### 2.6. Statistical Analysis

In a modified intention-to-treat approach, all participants who underwent randomization comprised the safety population, and those who provided at least one sample two weeks after receiving the intervention were regarded as the immunogenicity population. Means and proportions were used to report the baseline characteristics. We checked the homogeneity between the study groups by comparing the baseline characteristics. Geometric means for specific IgG and IgA antibody responses (presented as the Area under the curve, AUC) were calculated. Geometric mean ratios (GMR) and their 95% confidence interval for intranasal RCP compared to the placebo were estimated at day 14. We used independent and paired *t*-tests to examine between group and within group differences respectively. Subgroup analyses were conducted according to the time interval between the last vaccination and the intranasal booster dose, type of the last vaccine received, and type of the primary vaccination. The data were analyzed using Stata 14.2 (Stata Corporation, Texas, TX, USA), and *p*-values less than 0.05 were regarded as significant.

## 3. Results

**Figure 1:**
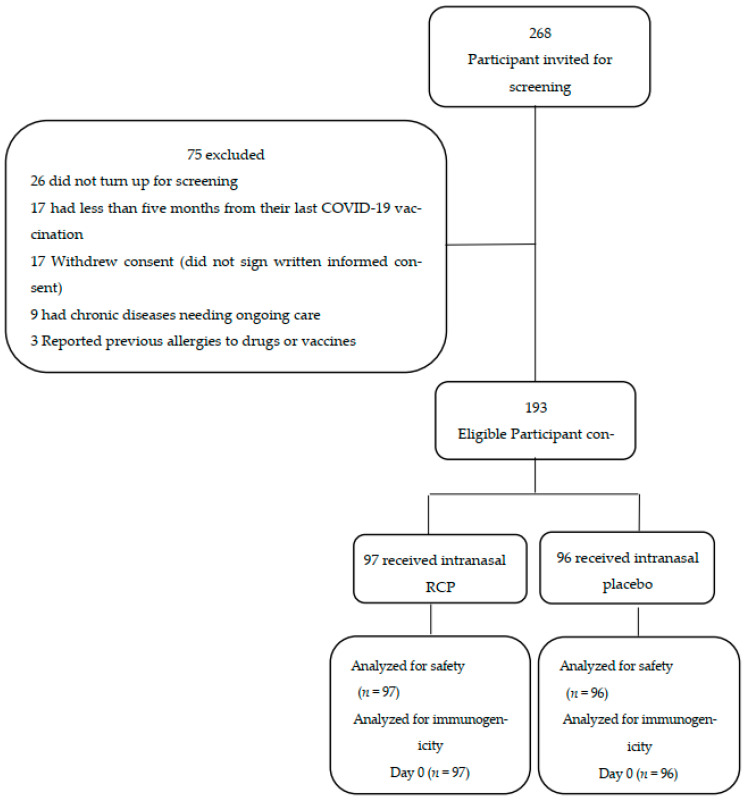
Participant’s flow diagram.

We enrolled 268 participants to enter the on-site screening process of which 193 eligible individuals were randomized into two groups of 97 and 96 people to receive the intranasal RCP rather than the primary vaccination or the placebo respectively (Figure 1). The mean age of the participants was 42 years and 73% were men (Table 1). Baseline comparison showed that the two groups are similar, indicating that an effective randomization has been achieved (Table 1).

**Figure 2:**
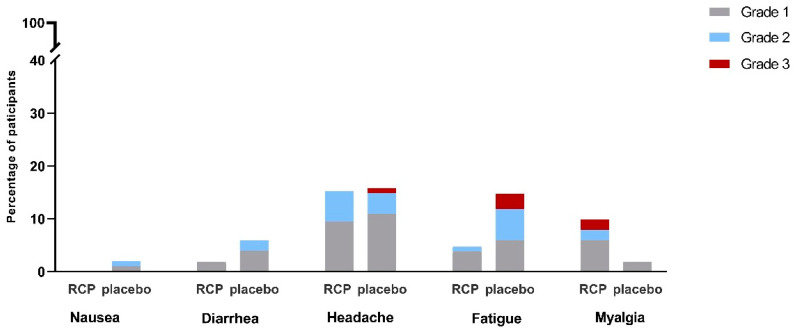
Frequency of solicited systemic adverse reactions by study groups during the first week following vaccination.

We observed no serious adverse reactions or anaphylaxis in the study participants. Solicited adverse reactions during the first week following the intervention were seen in 33 (34%) people in the vaccine and 41 (43%) people in the placebo groups, which was relatively similar (Table S2). Headache was the most prevalent solicited adverse reaction (16% in the vaccine vs. 16% in the placebo groups) followed by fatigue (5% vs. 15%) and myalgia (10% vs. 2%) (Figure 2). We detected 24 adverse events during 3150 person-days of follow-up in the intranasal RCP group (Incidence rate = 0.76%, 95%CI: 0.49–1.13), and with similar frequency, 33 events in 3030 person-days in the placebo group were detected (Incidence rate = 1.09%, 95%CI: 0.74–1.52). All observed adverse events resolved completely during the course of the study and were comparable in the two groups (Table S4). 

**Figure 3:**
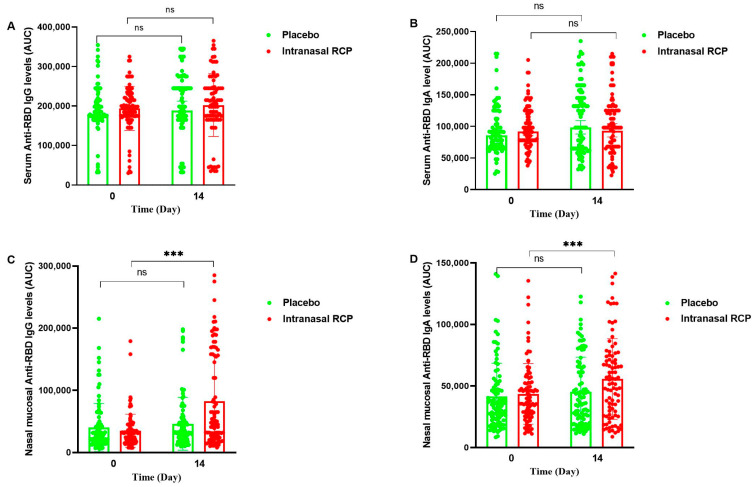
Scatter plots of individual values and their geometric means for specific IgG and IgA antibody levels in the serum and nasal mucosal secretions of the study participants at the time of booster IN vaccination and two weeks after. (**A**): Serum Anti-RBD IgG levels; (**B**): Serum Anti-RBD IgA levels; (**C**): Nasal mucosal Anti-RBD IgG levels; (**D**): Nasal mucosal Anti-RBD IgA levels. ns, not significant, * *p*  <  0.05, ** *p*  <  0.01, *** *p*  <  0.001.

**Figure 4:**
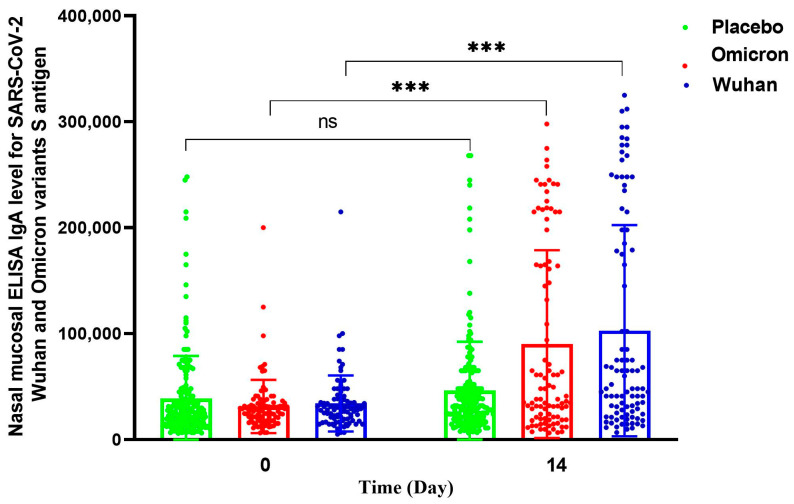
Scatter plots of individual values and their geometric means of nasal mucosal specific IgA anti-spike antibodies comparing the antibody response to Wuhan and Omicron variants in the study participants by study groups at the time of booster IN vaccination and two weeks after. ns, not significant, * *p*  <  0.05, ** *p*  <  0.01, *** *p*  <  0.001.

We observed a significant increase in the specific IgA (GMR = 1.26, 95% CI: 1.03–1.53) and IgG (GMR = 1.57, 95% CI: 1.22–2.01) anti-RBD antibodies in the nasal mucosa in the RCP group (Table 2, Figure 3), but their increase was not detectable in the serum and saliva samples (Figures S1–S3). Subgroup analyses by time interval between the last vaccination and the intranasal booster dose, by type of the last vaccine received, and by type of the primary vaccination yielded consistent results (Tables S8–S13, S15–S20 and S22–S24). Specific IgA anti-spike antibodies in the nasal mucosa also increased in the intranasal RCP group compared to the placebo. The increase against the COVID-19 variant Omicron (GMR = 1.72, 95% CI: 1.30–2.27) was also similar to that of Wuhan (GMR = 1.69, 95% CI: 1.29–2.22) (Figure 4 and Figures S4 and S5).

## 4. Discussion

Intranasal vaccines currently in use contain live attenuated viruses. One challenge with these vaccines is the potential risk of infection and transmission to the brain’s olfactory regions. However, employing a protein-based non-replicating type may be the optimal way to improve the safety of such vaccines [17,18,19,20]. The RCP intranasal vaccine is based on adjuvanted recombinant protein technology, and its use effectively eliminates this risk of infection. Additionally, RCP utilizes an adjuvant as a delivery system that can pass through the nasal epithelial cells and stimulate the immune system associated with this region [21].

We observed no serious adverse reactions or anaphylaxis in the specific study participants. The majority of the detected adverse reactions were self-limiting and all resolved completely, indicating a satisfactory safety profile. The specific IgA and IgG antibodies showed a significant increase in the nasal mucosal secretions of RCP recipients compared to the placebo indicating the ability of the vaccine to stimulate the NALT, while the same response was not seen in the serum. The immune responses seen towards the antigens derived from the Wuhan variant were replicated with similar magnitude towards the Omicron variant. 

We had lower participation of women compared to men in our study. However, comparison of the baseline information in the two study groups (Table 1) showed similar distributions of the baseline variables, indicative of an effective randomization process. Furthermore, we applied a rigorous blinding protocol on all specimens sent to the immunology lab that practically removes any possibility of differential treatments of the samples in the laboratory. Therefore, any observed differences between the immune responses in the two groups is unlikely to be due to the differences in the characteristics of the participants or acquisition of the information from their specimens, and most likely is a true effect of the intranasal RCP vaccine. 

We showed a statistically significant rise in specific IgA antibodies in the nasal mucosa of the participants who received the intranasal RCP vaccine compared with the placebo recipients (Table 2, Figure 3). This specific IgA antibody response was demonstrated in all subgroup analyses according to the interval between the last vaccination and the intranasal booster dose (5 to 9.5 months, 9.5 to 12.5 months, and 12.5 to 18 months), main types of the last vaccine received (Razi Cov Pars, Sinopharm and Spikogen), and main types of the primary vaccination (Razi Cov Pars, Sinopharm and AstraZeneca)—even though they did not reach statistical significance due to small numbers in the strata. Our phase I trial also indicated an increase in the specific IgA antibody response in the mucosa [9]. We attributed the stark phase I study response in the RCP vaccine group to the timing of the study (February 2021), which was in the early stages of the COVID-19 pandemic when the population’s cumulative exposure to various COVID-19 antigens had not happened yet. On the other hand, the general population today has had recurrent exposures over time to various COVID-19 variants; and therefore, the immune system in the upper respiratory tract mucosa has high baseline levels of secretory IgA, resulting in less dramatic responses to new antigenic exposures. The current study confirms that intranasal RCP can induce specific IgA responses in the upper respiratory tract mucosa. 

We also showed a statistically significant increase in IgG class antibodies in the nasal mucosa of the participants who received the intranasal RCP vaccine compared with the placebo recipients (Table 2, Figure 3D). This finding indicates that the intranasal RCP has been able to involve the immunological areas of the respiratory system beyond the nasal mucosa and nasal-associated lymphoid tissue (NALT). The presence of IgG antibodies in the nasal secretions is attributed to the induction of the specific IgG antibody in the lower areas, or possibly attributed to central immunity. It has proved that IgG, as part of the local airway host defense, acts in concert with IgA. It normally reaches the airways by transudation from plasma, but it can be added locally or directly in the airways as well [22,23].

Our findings on the Wuhan and Omicron variants (Figure 4) showed that the decrease in antibody specificity on other COVID-19 variants is much less than the IgA class antibody, and it can still function with high specificity for new variants [24].

One of our limitations in the current study is that we could not demonstrate the clinical efficacy of the observed increase in IgG and IgA class antibodies in the upper respiratory tract mucosa. Nor can we show that the spread of the virus has diminished following the administration of the intranasal dose. However, there is a substantial body of evidence that a rise in specific IgA class antibodies in the mucosa will result in improved mucosal protection [25]; and, therefore, it will have clinical implications [26]. Further studies with appropriate designs are needed to show the clinical impacts of the observed antibody changes. 

We replaced saliva samples with nasal swabs to assess the specific antibody responses in mucosal secretions. This decision was made for several reasons. One was the inherent difficulties in obtaining actual saliva samples from the participants. Furthermore, it is technically challenging to run valid ELISA antibody tests on saliva samples, given the influences of the microbial flora and various enzymes that can degrade proteins on the test results [14,26,27].

**Figure 5:**
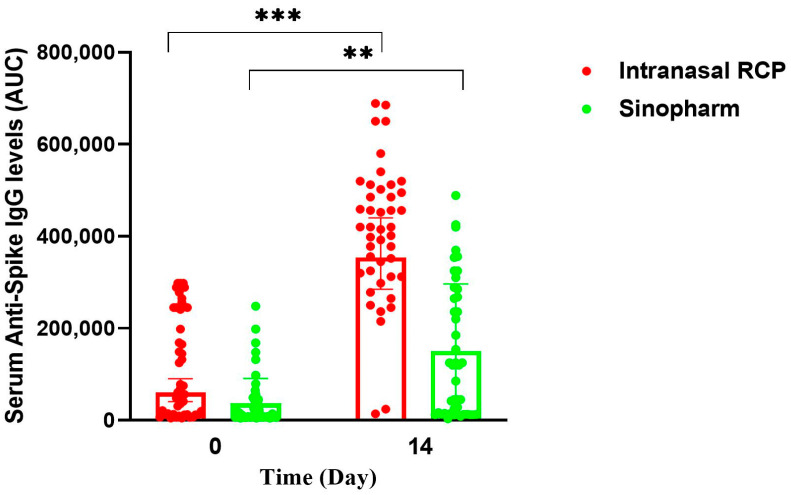
Scatter plots of individual values and their geometric means comparing the serum anti-Spike specific IgG in a subpopulation of phase III study participants in RAZI and Sinopharm groups in response to IN RCP or placebo (adjuvant), respectively, at the time of IN booster vaccination and two weeks after. * *p*  <  0.05, ** *p*  <  0.01, *** *p*  <  0.001.

We observed no rise in specific IgA and IgG antibodies in serum despite an increase in nasal mucosa (Table 2, Figure 3). This could be due to the participants already reaching peak serum antibody levels from their prior exposure to COVID-19, making it difficult to detect small differences at peak antibody levels. Nevertheless, we previously demonstrated as part of our phase III clinical trial that serum-specific anti-RBD IgA and anti-S IgG antibodies rose two weeks after the third intranasal dose in a subgroup of 100 participants (Figure 5 and Figures S4 and S5 from our recently published data) [11]. This rise may be a reflection of the participating subjects who had never received a COVID-19 vaccine before, hence having low levels of specific antibodies against COVID-19 antigens as well as the vaccination schedule that included two intramuscular doses just one month before the intranasal one. 

In summary, our findings here showed that the intranasal RCP vaccine as a booster dose has an acceptable safety profile and increases the IgA antibody in the nasal mucosal secretions compared to the placebo in all vaccine platforms, and this is statistically significant. Studies have shown that the increase of IgA antibodies in the nasal mucosa can play a role in reducing the transmission of the virus in the environment [28,29,30]. 

## 5. Conclusions

Intranasal RCP vaccine could serve as a potential booster on various vaccine platforms to improve mucosal protection. 

## Figures and Tables

**Table 1:** Baseline characteristics of the study participants.

	Placebo *n* = 96	Intranasal RCP *n* = 97	Total *n* = 193
Sex, *n* (%)			
Male	68 (70.8)	73 (75.3)	141 (73.1)
Female	28 (29.2)	24 (24.7)	52 (26.9)
Age group, *n* (%)			
<30 years.	17 (17.7)	11 (11.3)	28 (14.5)
30–50 years.	61 (63.5)	62 (63.9)	123 (63.7)
50–70 years.	18 (18.8)	22 (22.7)	40 (20.7)
>70 years.	0	2 (2.1)	2 (1.0)
Mean age, year (SD)	43.6 (12)	41.0 (11.3)	42.3 (11.7)
Mean Body Mass Index (SD)	26.7 (4.78)	26.4 (3.6)	26.5 (4.2)
Education, *n* (%)			
Under diploma	11 (11.5)	15 (15.5)	26 (13.5)
Diploma	38 (39.6)	36 (37.1)	74 (38.3)
Bachelor	28 (29.2)	31 (32.0)	59 (30.6)
Master and above	19 (19.8)	15 (15.5)	34 (17.6)
Vital signs, mean(min-max)			
Body temperature (°C) (min-max)	36.7 (35.8–37.8)	36.8 (35.7–37.1)	36.7 (35.7–37.8)
Heart rate (Per minute)	76.5 (55–108)	79.5 (63–108)	78.0 (55–108)
Respiratory rate (Per minute)	16.8 (14–19)	16.8 (14–20)	16.8 (14–20)
Systolic BP (mmHg)	114.0 (90–155)	115.2 (93–151)	114.6 (93–151)
Diastolic BP (mmHg)	76.5 (60–100)	77.4 (60–92)	77 (60–100)
Co-morbidities, *n* (%)			
Hypertension	4 (3.8)	7 (6.9)	11 (5.3)
Chronic heart diseases	0 (0.0)	1 (0.99)	1 (0.49)
Chronic kidney diseases	0 (0.0)	1 (0.99)	1 (0.49)
Mild liver diseases (fatty liver)	9 (8.5)	12 (11.9)	21 (10.2)
Diabetes with complications	5 (4.7)	6 (5.9)	11 (5.3)
Antibody levels (AUC), GM (95% CI)			
Serum anti-RBD IgG	179,016 (163,705–195,760)	181,381 (166,057–198,118)	180,214 (169,340–191,785)
Serum anti-RBD IgA	85,344 (78,607–92,659)	94,027 (86,399–102,328)	89,673 (84,543–95,115)
Nasal mucosal anti-RBD IgG	34,548 (30,461–39,185)	37,567 (33,529–42,090)	36,050 (33,141–39,214)
Nasal mucosal anti-RBD IgA	31,911 (31,016–32,831)	31,339 (30,648–32,046)	31,616 (31,057–32,185)
Time from the last vaccination, *n* (%)			
5–9.5 months	35 (36.5%)	30 (30.9%)	65 (33.7%)
9.5–12.5 months	33 (34.4%)	31 (31.9%)	64 (33.2%)
12.5–18 months	28 (29.2%)	36 (37.1%)	64 (33.1%)
Type of the last vaccine received, *n* (%)			
AstraZeneca (Viral vector)	4 (4.2)	4 (4.1)	8 (4.1)
Barakat (Inactivated)	1 (1.0)	1 (1.0)	2 (1.0)
Pastocovac (Protein subunit)	2 (2.1)	2 (2.1)	4 (2.1)
Razi Cov Pars (Protein subunit)	64 (66.7)	62 (63.9)	126 (65.3)
Sinopharm (Inactivated)	17 (17.7)	19 (19.6)	36 (18.6)
Spikogen (Protein subunit)	8 (8.3)	9 (9.3)	17 (8.8)

SD, Standard Deviation; GM, Geometric Mean; CI, Confidence Interval; AUC, Area Under the Curve. We prepared six serum dilutions (0·1, 0·01, 0·001, 0·0001, 0·00001, and 0·000001) for each serum sample to be tested by the ELISA method and calculated the area under the curve.

**Table 2:** Geometric Means (GM) and Geometric Mean Ratios (GMR) of specific IgG and IgA antibody levels in the serum and nasal mucosa of the study participants at the time of vaccination and two weeks after.

	Placebo	Intranasal RCP
Serum Anti-RBD IgG		
GM_AUC_ (95% CI)		
Baseline	179,016 (163,705–195,760, *n* = 91)	181,381 (166,058–198,119, *n* = 94)
Day 14	188,767 (167,867–212,269, *n* = 90)	178,344 (157,196–202,338, *n* = 86)
GMR_AUC_ (95% CI)		
Baseline	Ref	1.01 (0.89–1.15, *p* = 0.78)
Day 14	Ref	0.94 (0.80–1.12, *p* = 0.45)
Serum Anti-RBD IgA		
GM_AUC_ (95% CI)		
Baseline	85,344 (78,607–92,658, *n* = 91)	94,027.1 (86,399–102,328, *n* = 95)
Day 14	97,939 (87,859–109,177, *n* = 90)	93,169 (83,327–104,174, *n* = 86)
GMR_AUC_ (95% CI)		
Baseline	Ref	1.10 (0.98–1.24, *p* = 0.17)
Day 14	Ref	0.95 (0.81–1.11, *p* = 0.45)
Nasal mucosal Anti-RBD IgG		
GM_AUC_ (95% CI)		
Baseline	28,387 (24,135–33,373, *n* = 91)	28,558 (25,229–32,326, *n* = 94)
Day 14	34,302 (29,568–39794, *n* = 92)	53,724 (43,785–65,919, *n* = 90)
GMR_AUC_ (95% CI)		
Baseline	Ref	1.01 (0.82–1.23, *p* = 0.58)
Day 14	Ref	1.57 (1.22–2.01, *p* = 0.015)
Nasal mucosal Anti-RBD IgA		
GM_AUC_ (95% CI)		
Baseline	34,548 (30,461–39,185, *n* = 91)	37,567 (33,529–42,090, *n* = 94)
Day 14	37,159 (32,506–42,478, *n* = 92)	46,770 (40,346–54,217, *n* = 90)
GMR_AUC_ (95% CI)		
Baseline	Ref	1.09 (0.92–1.29, *p* = 0.29)
Day 14	Ref	1.26 (1.03–1.53, *p* = 0.046)

## Data Availability

Data is available within the article and in the supplementary file. Researchers who want to access individual participant data, documents, or other details of the project need to provide their request to the corresponding authors to approve and gain an agreement.

## Supplementary Materials

The following supporting information can be downloaded at: https://www.mdpi.com/article/10.3390/vaccines12111255/s1, Table S1: Comparison of post-intervention vital signs in study groups; Table S2: Number of systemic adverse events within the first week post-intervention in the study groups; Table S3: Incidence rate of unsolicited adverse events during the one month follow-up in the study groups; Table S4: Classification of all unsolicited adverse events during the one month follow-up in the study groups by ICD code; Table S5: List of all unsolicited adverse events during the one month follow-up in the study groups and their assessment regarding causal relationship; Table S6: Summary results of causality assessment of unsolicited adverse events during the one month follow-up in the study groups; Table S7: Geometric Means, Geometric Mean Ratio, and Geometric Mean Fold Increase for serum IgG antibodies against S antigen comparing intranasal RCP to the intranasal placebo; Table S8: Geometric Means, Geometric Mean Ratio, and Geometric Mean Fold Increase for serum IgG antibodies against S antigen comparing intranasal RCP to the intranasal placebo stratified by the time interval between last vaccination and the intranasal booster dose; Table S9: Geometric Means, Geometric Mean Ratio, and Geometric Mean Fold Increase for serum IgG antibodies against S antigen comparing intranasal RCP to the intranasal placebo stratified by type of last vaccine received; Table S10: Geometric Means, Geometric Mean Ratio, and Geometric Mean Fold Increase for serum IgG antibodies against S antigen comparing intranasal RCP to the intranasal placebo stratified by the type of the primary vaccination; Table S11: Geometric Means, Geometric Mean Ratio, and Geometric Mean Fold Increase for serum IgG antibodies against RBD antigen comparing intranasal RCP to the intranasal placebo stratified by the time interval between last vaccination and the intranasal booster dose; Table S12: Geometric Means, Geometric Mean Ratio, and Geometric Mean Fold Increase for serum IgG antibodies against RBD antigen comparing intranasal RCP to the intranasal placebo stratified by type of last vaccine received; Table S13: Geometric Means, Geometric Mean Ratio, and Geometric Mean Fold Increase for serum IgG antibodies against RBD antigen comparing intranasal RCP to the intranasal placebo stratified by the type of the primary vaccination; Table S14: Geometric Means, Geometric Mean Ratio, and Geometric Mean Fold Increase for serum IgA antibodies against S antigen comparing intranasal RCP to the intranasal placebo; Table S15: Geometric Means, Geometric Mean Ratio, and Geometric Mean Fold Increase for serum IgA antibodies against S antigen comparing intranasal RCP to the intranasal placebo stratified by the time interval between last vaccination and the intranasal booster dose; Table S16: Geometric Means, Geometric Mean Ratio, and Geometric Mean Fold Increase for serum IgA antibodies against S antigen comparing intranasal RCP to the intranasal placebo stratified by type of last vaccine received; Table S17: Geometric Means, Geometric Mean Ratio, and Geometric Mean Fold Increase for serum IgA antibodies against S antigen comparing intranasal RCP to the intranasal placebo stratified by the type of the primary vaccination; Table S18: Geometric Means, Geometric Mean Ratio, and Geometric Mean Fold Increase for serum IgA antibodies against S antigen comparing intranasal RCP to the intranasal placebo stratified by the time interval between last vaccination and the intranasal booster dose; Table S19: Geometric Means, Geometric Mean Ratio, and Geometric Mean Fold Increase for serum IgA antibodies against S antigen comparing intranasal RCP to the intranasal placebo stratified by type of last vaccine received; Table S20: Geometric Means, Geometric Mean Ratio, and Geometric Mean Fold Increase for serum IgA antibodies against S antigen comparing intranasal RCP to the intranasal placebo stratified by the type of the primary vaccination; Table S21: Geometric Means, Geometric Mean Ratio, and Geometric Mean Fold Increase for saliva IgA antibodies against RBD antigen comparing intranasal RCP to the intranasal placebo; Table S22: Geometric Means, Geometric Mean Ratio, and Geometric Mean Fold Increase for nasal mucosal IgA antibodies against RBD antigen comparing intranasal RCP to the intranasal placebo stratified by the time interval between last vaccination and the intranasal booster dose; Table S23: Geometric Means, Geometric Mean Ratio, and Geometric Mean Fold Increase for nasal mucosal IgA antibodies against RBD antigen comparing intranasal RCP to the intranasal placebo stratified by type of last vaccine received; Table S24: Geometric Means, Geometric Mean Ratio, and Geometric Mean Fold Increase for nasal mucosal IgA antibodies against RBD antigen comparing intranasal RCP to the intranasal placebo stratified by the type of the primary vaccination; Table S25: Geometric Means, Geometric Mean Ratio, and Geometric Mean Fold Increase for nasal mucosal IgA antibodies against RBD antigen from the Omicron variant of SARS-CoV-2 comparing intranasal RCP to the intranasal placebo; Table S26: Geometric Means, Geometric Mean Ratio, and Geometric Mean Fold Increase for nasal mucosal IgA antibodies against RBD antigen from the Wuhan variant of SARS-CoV-2 comparing intranasal RCP to the intranasal placebo; Figure S1: Scatter plots of individual values and their geometric means for specific IgG antibody levels against S antigen in the serum at the time of vaccination and two weeks after by study groups, Figure S2: Scatter plots of individual values and their geometric means for specific IgA antibody levels against S antigen in the serum at the time of vaccination and two weeks after by study groups, Figure S3: Scatter plots of individual values and their geometric means for specific IgA antibody levels against RBD antigen in the saliva at the time of vaccination and two weeks after by study groups, Figure S4: Scatter plots of individual values and their geometric means for specific IgA antibody levels against RBD antigen from the Omicron variant of SARS-CoV-2 in the nasal mucosa at the time of vaccination and two weeks after by study groups, Figure S5: Scatter plots of individual values and their geometric means for specific IgA antibody levels against RBD antigen from the Wuhan variant of SARS-CoV-2 in the nasal mucosa at the time of vaccination and two weeks after by study groups, Figure S6: Scatter plots of individual values and their geometric means of anti-RBD specific IgA antibody in saliva of a subpopulation of phase III study participants in RAZI and Sinopharm groups in response to IN RCP or placebo, respectively, at the time of IN booster vaccination and two weeks later, Figure S7: Scatter plots of individual values and their geometric means of anti-RBD specific IgA antibody in serum of a subpopulation of phase III study participants in RAZI and Sinopharm groups in response to IN RCP or placebo, respectively, at the time of IN booster vaccination and two weeks later.
