# Supplementary material for: Safety and Immunogenicity of Intranasal Razi Cov Pars as a COVID-19 Booster Vaccine in Adults: Promising Results from a Groundbreaking Clinical Trial
Source: Vaccines (Basel). 2024 Nov 5;12(11):1255. doi: 10.3390/vaccines12111255 (PMC11598910; doi:10.3390/vaccines12111255)

Supplement file

Safety and Immunogenicity of Intranasal Razi Cov Pars as a COVID-19 Booster Vaccine in Adults: Promising Results from a Groundbreaking Clinical Trial

Table of Content

Contents

Table of Content 1

Safety outcomes **4**

Post-intervention vital signs 4

Table S1 Comparison of post-intervention vital signs in study groups 4

Solicited systemic adverse events within the first week post-intervention 4

Table S2 Number of Systemic adverse event within the first week post-intervention in the study groups 4

Unsolicited adverse events 4

Table S3 Incidence rate of unsolicited adverse events during one month follow-up in the study groups 4

Table S4 Classification of all unsolicited adverse events during one month follow-up in the study groups by ICD code 4

Table S5 List of all unsolicited adverse events during one month follow-up in the study groups and their assessment regarding causal relationship 5

Table S6 Summary results of causality assessment of unsolicited adverse events during one month follow-up in the study groups 7

Efficacy outcomes **8**

Serum ELISA IgG level for SARS-CoV-2 S-antigen 8

Table S7 Geometric means, Geometric Mean Ratio and Geometric Mean Fold Increase for serum IgG antibodies against S antigen comparing intranasal RCP to the intranasal placebo 8

Figure S1 Scatter plots of individual values and their geometric means for specific IgG antibody levels against S antigen in the serum at the time of vaccination and two weeks after by study groups 8

Table S8 Geometric means, Geometric Mean Ratio and Geometric Mean Fold Increase for serum IgG antibodies against S antigen comparing intranasal RCP to the intranasal placebo stratified by the time interval between last vaccination and the intranasal booster dose 9

Table S9 Geometric means, Geometric Mean Ratio and Geometric Mean Fold Increase for serum IgG antibodies against S antigen comparing intranasal RCP to the intranasal placebo stratified by type of last vaccine received 9

Table S10 Geometric means, Geometric Mean Ratio and Geometric Mean Fold Increase for serum IgG antibodies against S antigen comparing intranasal RCP to the intranasal placebo stratified by the type of the primary vaccination 10

Serum ELISA IgG level for SARS-CoV-2 RBD-antigen *11*

Table S11 Geometric means, Geometric Mean Ratio and Geometric Mean Fold Increase for serum IgG antibodies against RBD antigen comparing intranasal RCP to the intranasal placebo stratified by the time interval between last vaccination and the intranasal booster dose 11

Table S12 Geometric means, Geometric Mean Ratio and Geometric Mean Fold Increase for serum IgG antibodies against RBD antigen comparing intranasal RCP to the intranasal placebo stratified by type of last vaccine received 11

Table S13 Geometric means, Geometric Mean Ratio and Geometric Mean Fold Increase for serum IgG antibodies against RBD antigen comparing intranasal RCP to the intranasal placebo stratified by the type of the primary vaccination 12

Serum ELISA IgA level for SARS-CoV-2 S-antigen *13*

Table S14 Geometric means, Geometric Mean Ratio and Geometric Mean Fold Increase for serum IgA antibodies against S antigen comparing intranasal RCP to the intranasal placebo 13

Figure S2 Scatter plots of individual values and their geometric means for specific IgA antibody levels against S antigen in the serum at the time of vaccination and two weeks after by study groups 13

Table S15 Geometric means, Geometric Mean Ratio and Geometric Mean Fold Increase for serum IgA antibodies against S antigen comparing intranasal RCP to the intranasal placebo stratified by the time interval between last vaccination and the intranasal booster dose 14

Table S16 Geometric means, Geometric Mean Ratio and Geometric Mean Fold Increase for serum IgA antibodies against S antigen comparing intranasal RCP to the intranasal placebo stratified by type of last vaccine received 14

Table S17 Geometric means, Geometric Mean Ratio and Geometric Mean Fold Increase for serum IgA antibodies against S antigen comparing intranasal RCP to the intranasal placebo stratified by the type of the primary vaccination 15

Serum ELISA IgA level for SARS-CoV-2 RBD-antigen *16*

Table S18 Geometric means, Geometric Mean Ratio and Geometric Mean Fold Increase for serum IgA antibodies against S antigen comparing intranasal RCP to the intranasal placebo stratified by the time interval between last vaccination and the intranasal booster dose 16

Table S19 Geometric means, Geometric Mean Ratio and Geometric Mean Fold Increase for serum IgA antibodies against S antigen comparing intranasal RCP to the intranasal placebo stratified by type of last vaccine received 16

Table S20 Geometric means, Geometric Mean Ratio and Geometric Mean Fold Increase for serum IgA antibodies against S antigen comparing intranasal RCP to the intranasal placebo stratified by the type of the primary vaccination 17

Saliva ELISA IgA level for SARS-CoV-2 RBD-antigen *18*

Table S21 Geometric means, Geometric Mean Ratio and Geometric Mean Fold Increase for saliva IgA antibodies against RBD antigen comparing intranasal RCP to the intranasal placebo 18

Figure S3 Scatter plots of individual values and their geometric means for specific IgA antibody levels against RBD antigen in the saliva at the time of vaccination and two weeks after by study groups 18

Nasal mucosal ELISA IgA level for SARS-CoV-2 RBD-antigen 19

Table S22 Geometric means, Geometric Mean Ratio and Geometric Mean Fold Increase for nasal mucosal IgA antibodies against RBD antigen comparing intranasal RCP to the intranasal placebo stratified by the time interval between last vaccination and the intranasal booster dose 19

Table S23 Geometric means, Geometric Mean Ratio and Geometric Mean Fold Increase for nasal mucosal IgA antibodies against RBD antigen comparing intranasal RCP to the intranasal placebo stratified by type of last vaccine received 19

Table S24 Geometric means, Geometric Mean Ratio and Geometric Mean Fold Increase for nasal mucosal IgA antibodies against RBD antigen comparing intranasal RCP to the intranasal placebo stratified by the type of the primary vaccination 20

Nasal mucosal ELISA IgA level for Omicron variant of SARS-CoV-2 S-antigen 21

Table S25 Geometric means, Geometric Mean Ratio and Geometric Mean Fold Increase for nasal mucosal IgA antibodies against RBD antigen from Omicron variant of SARS-CoV-2 comparing intranasal RCP to the intranasal placebo 21

Figure S4 Scatter plots of individual values and their geometric means for specific IgA antibody levels against RBD antigen from Omicron variant of SARS-CoV-2 in the nasal mucosa at the time of vaccination and two weeks after by study groups 21

Nasal mucosal ELISA IgA level for Wuhan variant of SARS-CoV-2 S-antigen 22

Table S26 Geometric means, Geometric Mean Ratio and Geometric Mean Fold Increase for nasal mucosal IgA antibodies against RBD antigen from Wuhan variant of SARS-CoV-2 comparing intranasal RCP to the intranasal placebo 22

Figure S5 Scatter plots of individual values and their geometric means for specific IgA antibody levels against RBD antigen from Wuhan variant of SARS-CoV-2 in the nasal mucosa at the time of vaccination and two weeks after by study groups 22

Results for a subgroup of 100 participants from Phase III clinical trial study 23

Saliva and serum IgA level for SARS-CoV-2 S antigen 23

Figure S6 Scatter plots of individual values and their geometric means of anti-RBD specific IgA antibody in saliva of a subpopulation of phase III study participants in RAZI and Sinopharm groups in response to IN RCP or placebo, respectively, at the time of IN booster vaccination and two weeks later 23

Figure S7 Scatter plots of individual values and their geometric means of anti-RBD specific IgA antibody in serum of a subpopulation of phase III study participants in RAZI and Sinopharm groups in response to IN RCP or placebo, respectively, at the time of IN booster vaccination and two weeks later 24

Safety outcomes

Post-intervention vital signs

Table S1 Comparison of post-intervention vital signs in study groups

	Intranasal RCP n=97	Placebo n=96	Total n=193
Body temperature (°C)	36.8 (36.2 – 37.2	36.7(35.7 – 37.3)	36.8(35.7 – 37.7)
Heart rate (Per minute)	75.4(57 - 98)	75.2(57 - 100)	75.3(57 - 100)
Respiratory rate (Per minute)	16.7(14 - 19)	16.6(14 - 20)	16.6(14 - 20)
Systolic BP (mmHg)	114.5(88 - 150)	112.1(85-144)	113.4(85 - 150)
Diastolic BP (mmHg)	77.2(61 - 95)	76.2(60 - 96)	76.7(60-96)

Solicited systemic adverse events within the first week post-intervention

Table S2 Number of Systemic adverse event within the first week post-intervention in the study groups

		**Intranasal RCP n=97**	**Placebo n=96**
**Nausea**			
	Grade 1	0(0)	1(0.99)
	Grade 2	0(0)	1(0.99)
**Diarrhea**			
	Grade 1	2(1.90)	4(3.96)
	Grade 2	0(0)	2(1.98)
**Headache**			
	Grade 1	10(9.52)	11(10.89)
	Grade 2	6(5.71)	4(3.96)
	Grade 3	0(0)	1(0.99)
**Fatigue**			
	Grade 1	4(3.81)	6(5.94)
	Grade 2	1(0.95)	6(5.94)
	Grade 3	0(0)	3(2.97)
**Myalgia**			
	Grade 1	6 (5.94)	2(1.90)
	Grade 2	2 (1.98)	0 (0.0)
	Grade 3	2 (1.98)	0 (0.0)

Unsolicited adverse events

Table S3 Incidence rate of unsolicited adverse events during one month follow-up in the study groups

Group	Person time	Event	Incidence Rate percent (95 % CI)
Intranasal RCP	3150	24	0.79 (0.51 – 1.17)
Placebo	3030	33	1.09 (0.74 – 1.52)

Table S4 Classification of all unsolicited adverse events during one month follow-up in the study groups by ICD code

ICD-10 code	ICD Description	In-RCP	Placebo	Total
B34.9	Viral infection, unspecified B34.9	6 (0.19)	13 (0.43)	19 (0.31)
R42	Dizziness and giddiness R42	2 (0.06)	2 (0.07)	4 (0.06)
J02	Acute pharyngitis J02	2 (0.06)	1 (0.03)	3 (0.05)
H66.9	Otitis media, unspecified H66.9	0 (0.00)	2 (0.07)	2 (0.03)

J06.9	Acute upper respiratory infection, unspecified	1 (0.03)	1 (0.03)	2 (0.03)
J39.9	Disease of upper respiratory tract, unspecified	0 (0.00)	2 (0.07)	2 (0.03)
M54.9	Dorsalgia, unspecified M54.9	0 (0.00)	2 (0.07)	2 (0.03)
M79.9	Soft tissue disorder, unspecified M79.9	2 (0.06)	0 (0.00)	2 (0.03)
R23.8	Other and unspecified skin changes R23.8	0 (0.00)	2 (0.07)	2 (0.03)
A09.9	Gastroenteritis and colitis of unspecified origin	0 (0.00)	1 (0.03)	1 (0.02)
B00.9	Herpes viral infection, unspecified B00.9	0 (0.00)	1 (0.03)	1 (0.02)
B39.4	Histoplasmosis capsulati, unspecified B39.4	1 (0.03)	0 (0.00)	1 (0.02)
J02.9	Acute pharyngitis, unspecified J02.9	1 (0.03)	0 (0.00)	1 (0.02)
K14.9	Disease of tongue, unspecified K14.9	1 (0.03)	0 (0.00)	1 (0.02)
K42	Umbilical hernia K42	0 (0.00)	1 (0.03)	1 (0.02)
M54	Dorsalgia M54	0 (0.00)	1 (0.03)	1 (0.02)
M79.1	Myalgia M79.1	0 (0.00)	1 (0.03)	1 (0.02)
M79.6	Pain in limb M79.6	0 (0.00)	1 (0.03)	1 (0.02)
N39.0	Urinary tract infection, site not specified N39.0	1 (0.03)	0 (0.00)	1 (0.02)
N73.9	Female pelvic inflammatory disease, unspecified N73.9	1 (0.03)	0 (0.00)	1 (0.02)
R03.0	Elevated blood-pressure reading, without diagnosis of hypertension R03.0	1 (0.03)	0 (0.00)	1 (0.02)
R04.9	Haemorrhage from respiratory passages, unspecified R04.9	0 (0.00)	1 (0.03)	1 (0.02)
R05	Cough R05	1 (0.03)	0 (0.00)	1 (0.02)
R10.4	Other and unspecified abdominal pain R10.4	1 (0.03)	0 (0.00)	1 (0.02)
R35	Polyuria R35	1 (0.03)	0 (0.00)	1 (0.02)
R51	Headache R51	1 (0.03)	0 (0.00)	1 (0.02)
R52	Pain, not elsewhere classified R52	1 (0.03)	0 (0.00)	1 (0.02)
U07.2	Emergency use of U07.2	0 (0.00)	1 (0.03)	1 (0.02)
Total		24 (0.79)	33 (1.09)	57 (0.94)

Table S5 List of all unsolicited adverse events during one month follow-up in the study groups and their assessment regarding causal relationship

ID	Intervention date (YMD)	Adverse event date (YMD)	Intervention type	Symptom and signs	Causal relationship
930	2023/1/9	2023/1/10	Intranasal RCP	Tongue bolus	Un-assessable
892	2023/1/9	2023/1/10	Intranasal RCP	Knee pain	suspicious
533	2023/1/3	2023/1/10	Placebo	Low back pain	Un-assessable
533	2023/1/3	2023/1/30	Placebo	Diziness	Un-assessable
629	2023/1/10	2023/1/13	Placebo	Epistaxis	unrelated
629	2023/1/10	2023/2/5	Placebo	Common cold symptoms	Un-assessable
978	2023/1/9	2023/1/11	Intranasal RCP	Headache	probable
978	2023/1/9	2023/2/11	Intranasal RCP	Sore throat, fatigue	unrelated
536	2023/1/7	2023/1/8	Placebo	Headache, fatigue	unrelated
912	2023/1/10	2023/1/10	Placebo	Chill and fever, chest pain	unrelated
682	2023/1/10	2023/1/24	Placebo	Knee pain, myaligia	unlikely

468	2023/1/22	2023/1/24	Placebo	Epistaxis	probable
453	2023/1/23	2023/1/23	Intranasal RCP	Hypertension, headache	probable
453	2023/1/23	2023/2/8	Intranasal RCP	Rinhorea, prespiration	unrelated
251	2023/1/24	2023/1/28	Placebo	dry cough, fatigue, rinhorea	unrelated
169	2023/1/9	2023/1/9	Intranasal RCP	Sore throat, rinhorea	unrelated
666	2023/1/15	2023/1/30	Intranasal RCP	Swollen ankle	unrelated
666	2023/1/15	2023/2/27	Intranasal RCP	Skin rash	unrelated
980	2023/1/18	2023/1/27	Placebo	Herpes simplex	unrelated
458	2023/1/10	2023/1/12	Placebo	Low back pain	Un-assessable
458	2023/1/10	2023/2/5	Placebo	Dizziness	Un-assessable
354	2023/1/21	2023/2/1	Intranasal RCP	Mild sore throat	unrelated
984	2023/1/29	2023/1/31	Intranasal RCP	Back pain, lack of energy	unrelated
684	2023/1/4	2023/1/7	Placebo	Muscle pain and cramp	unrelated
927	2023/1/9	2023/2/1	Intranasal RCP	Fever and lack of energy	unrelated
873	2023/1/7	2023/1/23	Placebo	Rinhora, Ear fullness	unrelated
112	2023/1/17	2023/2/5	Placebo	Body pain and heavy breathing	unrelated
255	2023/1/29	2023/2/3	Placebo	Diarrhea	unrelated
931	2023/1/23	2023/2/5	Placebo	Urinary frquency	Un-assessable
562	2023/1/24	2023/2/1	Intranasal RCP	Dizziness	unrelated
990	2023/1/9	2023/1/12	Intranasal RCP	Dry cough	Un-assessable
107	2023/1/23	2023/2/6	Intranasal RCP	Sore throat	unrelated
746	2023/1/24	2023/2/8	Placebo	Headache, Sore throat	unrelated
145	2023/1/31	2023/2/7	Placebo	Rinhorea, body pain	unrelated
265	2023/2/7	2023/2/10	Intranasal RCP	Dry cough	unrelated
375	2023/1/10	2023/1/18	Placebo	Cough and post nasal discharge	unrelated
961	2023/1/25	2023/2/12	Placebo	Back pain	unrelated
635	2023/1/23	2023/2/13	Placebo	Nausea, Sore throat	unrelated
133	2023/1/31	2023/2/12	Placebo	Sore throat, body pain	unrelated
570	2023/2/8	2023/2/9	Placebo	Skin rash on chest and face	probable
256	2023/2/1	2023/2/7	Placebo	Sore throat	unrelated
256	2023/2/1	2023/2/28	Placebo	Umbilical hernia	unrelated
593	2023/1/28	2023/2/8	Placebo	Common cold symptoms	unrelated
441	2023/1/29	2023/2/15	Placebo	Sore throat, congested nose	unrelated
180	2023/2/15	2023/2/17	Placebo	Fever, body pain	unrelated
180	2023/2/15	2023/3/20	Placebo	Body pain	unrelated
241	2023/2/14	2023/2/14	Intranasal RCP	Sore throat, rinhora	unrelated
628	2023/2/14	2023/2/27	Intranasal RCP	Dizziness	unrelated
715	2023/1/9	2023/2/22	Intranasal RCP	Headache	unrelated
320	2023/1/29	2023/2/21	Placebo	Headache and lack of energy	unrelated
731	2023/2/22	2023/2/22	Intranasal RCP	Dizziness	probable
681	2023/2/13	2023/3/8	Placebo	Body pain, headache	unrelated
639	2023/2/15	2023/3/11	Placebo	Fever and chill	unrelated
728	2023/1/9	2023/1/12	Placebo	Itsching erythematous rash	probable
679	2023/1/10	2023/1/11	Placebo	Diarrhea and headache	probable
475	2023/2/22	2023/3/15	Intranasal RCP	Urinary infection	unrelated
475	2023/2/22	2023/3/22	Intranasal RCP	Fever, loss of taste and smell	unrelated

Table S6 Summary results of causality assessment of unsolicited adverse events during one month follow-up in the study groups

	In-RCP	Placebo	Total
Not Related	17	23	40
Unlikely	0	1	1
Suspected/Possible	1	0	1
Probable	3	4	7
Not assessable	3	5	8
Total	24	33	57

Efficacy outcomes

Serum ELISA IgG level for SARS-CoV-2 S-antigen

Table S7 Geometric means, Geometric Mean Ratio and Geometric Mean Fold Increase for serum IgG antibodies against S antigen comparing intranasal RCP to the intranasal placebo

	Placebo	Intranasal RCP
GM_{AUC} (95% CI)		
Baseline	199827.1 (184466-216467, n=91)	202164.4 (187356.8 – 218102.3, n=95)
Day 14	209188.8 (188454.3-232204.6, n=93)	195423.7 (173907.6 – 219601.9, n=91)
$GMFI_{AUC}$ (95% CI)		
Baseline	1 (Reference)	1 (Reference)
Day 14	1.03 (0.94-1.14, n=88)	0.96 (0.85-1.09, n=89)
GMR_{AUC} (95% CI)		
Baseline	1 (Reference)	1.01 (0.91-1.13)
Day 14	1 (Reference)	0.93 (0.80-1.08)

Figure S1 Scatter plots of individual values and their geometric means for specific IgG antibody levels against S antigen in the serum at the time of vaccination and two weeks after by study groups

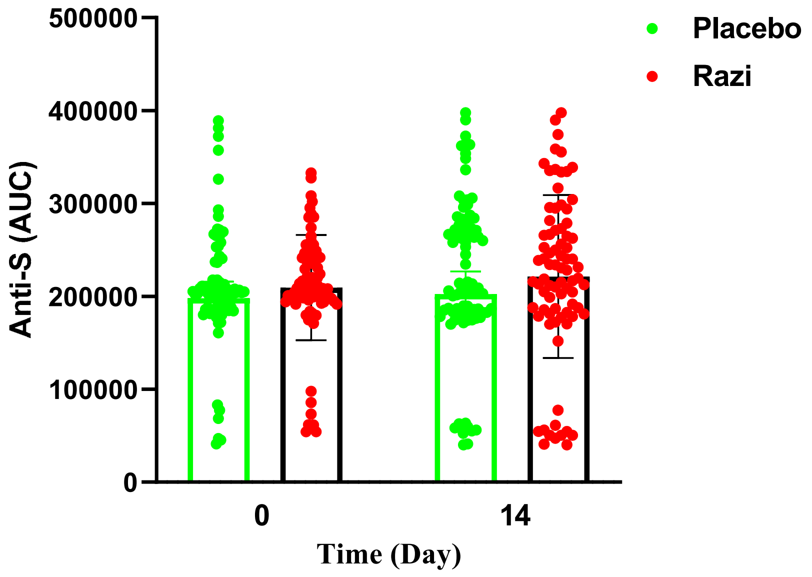

Table S8 Geometric means, Geometric Mean Ratio and Geometric Mean Fold Increase for serum IgG antibodies against S antigen comparing intranasal RCP to the intranasal placebo stratified by the time interval between last vaccination and the intranasal booster dose

	**Tertile 1, n=65 (5 – 9.5 month)**	**Tertile 2, n=64 (9.5 – 12.5 months)**	**Tertile 3, n=64 (12.5 – 18 months)**	**P trend**
GM_{AUC} (95% CI)				
Baseline, placebo group	205591.0 (175832.6-240385.9, n=34)	213123.0 (190854.9-235391.1, n=29)	198206.0 (179109.4-217302.5, n=28)	0.33
Day 14, placebo group	217582.4 (178205.3-265660.4, n=34)	239006.8 (211665.9-266347.8, n=33)	201439.2 (172139.7-230738.7, n=26)	0.10
Baseline , RCP group	204364.9 (180815.6-227914.2, n=30)	208539.2 (187559.5-229518.9, n=29)	216510.6 (195356.0-239956.0, n=36)	0.31
Day 14, RCP group	226023.7 (196706.6-255340.8, n=28)	221109.9 (188390.2-253829.5, n=30)	183975.5 (147146.9-230021.8, n=33)	0.83
$GMFI_{AUC}$ (95% CI)				
Reference (Baseline)	1.00	1.00	1.00	
Day 14, placebo group	1.1 (0.9-1.3, n=33)	1.1 (0.9-1.3, n=29)	1.0 (0.9-1.1, n=26)	0.50
Day 14, RCP group	1.1 (0.9-1.4, n=28)	1.0 (0.8-1.2, n=28)	0.8 (0.7-1.1, n=33)	0.28
GMR_{AUC} (95% CI)				
Placebo group	1 (Reference)	1 (Reference)	1 (Reference)	
RCP group	0.96 (0.73-1.25)	0.88 (0.69-1.13)	1.01 (0.74-1.37)	0.84

Table S9 Geometric means, Geometric Mean Ratio and Geometric Mean Fold Increase for serum IgG antibodies against S antigen comparing intranasal RCP to the intranasal placebo stratified by type of last vaccine received

	**Razi Cov Pars**	**Sinopharm**	**Spikogen**	**P trend**
GM_{AUC} (95% CI)				
Baseline, placebo group	208728.3 (193875.9-224718.4, n=61)	141273.3 (104456.9-191065.9, n=16)	289484.5 (230922.2-362898.3, n=7)	<0.001
Day 14, placebo group	224369.9 (199093.1-252855.8, n=61)	176110.9 (130250.8-238117.8, n=17)	243003.1 (187076.2-315649.4, n=8)	0.097
Baseline , RCP group	205826.5 (188687.9-224521.9, n=61)	172949.0 (134176.1-222926.0, n=19)	241073.2 (206001.4-282115.9, n=8)	0.081
Day 14, RCP group	193764.7 (166937.7-224902.8, n=59)	187498.1 (148899.6-236102.2, n=19)	207169.3 (103582.1-414348.9, n=7)	0.56
$GMFI_{AUC}$ (95% CI)				
Reference (Baseline)	1.00	1.00	1.00	
Day 14, placebo group	0.9 (0.8-1.1, n=58)	1.2 (0.9-1.7, n=16)	0.9 (0.7-1.0, n=7)	0.09
Day 14, RCP group	1.1 (0.8-1.4, n=19)	1.1 (0.8-1.4, n=19)	0.8 (0.4-1.6, n=6)	0.51
GMR_{AUC} (95% CI)				
Placebo group	1.00	1.00	1.00	
RCP group	0.86 (0.72-1.04)	1.06 (0.74-1.53)	0.85 (0.46-1.59)	0.67

Table S10 Geometric means, Geometric Mean Ratio and Geometric Mean Fold Increase for serum IgG antibodies against S antigen comparing intranasal RCP to the intranasal placebo stratified by the type of the primary vaccination

	**Razi Cov Pars**	**Sinopharm**	**Spikogen**	**P trend**
GM_{AUC} (95% CI)				
Baseline, placebo group	211378.6 (195394.0-228670.9, n=58)	178019.5 (144007.0-220065.4, n=25)	170871.6 (96273.4-303272.7, n=5)	0.16
Day 14, placebo group	228161.5 (205187.9-253707.4, n=59)	172624.7 (133327.9-223503.8, n=26)	223100.6 (170709.4-291570.7, n=5)	0.08
Baseline , RCP group	203004.1 (186102.7-221440.4, n=60)	188273.6 (153790.5-230488.6, n=25)	215254.0 (145810.8-317770.0, n=4)	0.84
Day 14, RCP group	182744.6 (155448.7-214833.4, n=58)	200852.6 (165405.7-243895.9, n=24)	259707.3 (128588.1-524526.4, n=3)	0.52
$GMFI_{AUC}$ (95% CI)				
Reference (Baseline)	1.00	1.00	1.00	
Day 14, placebo group	1.1 (1.0-1.2, n=55)	1.0 (0.7-1.2, n=25)	1.3 (0.6-2.7, n=5)	0.50
Day 14, RCP group	0.9 (0.8-1.1, n=57)	1.1 (0.9-1.4, n=23)	1.2 (0.8-1.6, n=3)	0.69
GMR_{AUC} (95% CI)				
Placebo group	1 (Reference)	1 (Reference)	1 (Reference)	
RCP group	0.80 (0.66-0.97)	1.16 (0.85-1.60)	1.16 (0.76-1.79)	0.057

Serum ELISA IgG level for SARS-CoV-2 RBD-antigen

Table S11 Geometric means, Geometric Mean Ratio and Geometric Mean Fold Increase for serum IgG antibodies against RBD antigen comparing intranasal RCP to the intranasal placebo stratified by the time interval between last vaccination and the intranasal booster dose

	**Tertile 1, n=65 (5 – 9.5 month)**	**Tertile 2, n=64 (9.5 – 12.5 months)**	**Tertile 3, n=64 (12.5 – 18 months)**	**P trend**
GM_{AUC} (95% CI)				
Baseline, placebo group	183313.0 (154850.0-217007.7, n=34)	185098.7 (161978.9-211518.4, n=29)	168017.9 (141583.8-199387.3, n=28)	0.38
Day 14, placebo group	197922.7 (157668.1-248454.9, n=32)	201495.3 (171592.4-236609.3, n=32)	164336.0 (129419.6-208672.7, n=26)	0.11
Baseline , RCP group	165222.9 (136553.2-199911.9, n=30)	173341.2 (143515.2-209365.9, n=29)	204004.8 (187647.3-221788.4, n=35)	0.09
Day 14, RCP group	181311.5 (144722.7-227150.7, n=25)	188887.1 (154063.2-231582.6, n=29)	167130.7 (131134.2-213008.3, n=32)	0.83
$GMFI_{AUC}$ (95% CI)				
Reference (Baseline)	1.00	1.00	1.00	
Day 14, placebo group	1.1 (0.8-1.3, n=31)	1.1 (0.9-1.3, n=29)	1.0 (0.9-1.1, n=26)	0.22
Day 14, RCP group	1.1 (0.8-1.4, n=25)	1.1 (0.9-1.3, n=27)	0.8 (0.6-1.0, n=31)	0.51
GMR_{AUC} (95% CI)				
Placebo group	1 (Reference)	1 (Reference)	1 (Reference)	
RCP group	0.92 (0.67-1.26)	0.94 (0.73-1.21)	1.02 (0.73-1.42)	0.67

Table S12 Geometric means, Geometric Mean Ratio and Geometric Mean Fold Increase for serum IgG antibodies against RBD antigen comparing intranasal RCP to the intranasal placebo stratified by type of last vaccine received

	**Razi Cov Pars**	**Sinopharm**	**Spikogen**	**P trend**
GM_{AUC} (95% CI)				
Baseline, placebo group	187981.1 (173250.3-203964.5, n=61)	121941.0 (85843.8-173217.0, n=16)	262464.6 (209730.1-328458.6, n=7)	<0.001
Day 14, placebo group	202047.5 (176304.7-231549.2, n=59)	159120.6 (112034.4-225996.4, n=16)	223228.6 (175376.6-284137.2, n=8)	0.16
Baseline , RCP group	183107.6 (165354.2-202767.2, n=61)	158359.1 (116160.2-215888.1, n=18)	215630.6 (181444.1-256258.3, n=8)	0.25
Day 14, RCP group	172610.2 (145796.7-204354.9, n=55)	168603.8 (129260.1-219922.7, n=19)	250004.6 (204545.9-305566.0, n=6)	0.18
$GMFI_{AUC}$ (95% CI)				
Reference (Baseline)	1 (Reference)	1 (Reference)	1 (Reference)	
Day 14, placebo group	1.1 (0.9-1.2, n=57)	1.3 (0.9-1.9, n=15)	0.9 (0.7-1.0, n=7)	0.048
Day 14, RCP group	0.9 (0.8-1.1, n=54)	1.1 (0.8-1.4, n=18)	1.1 (0.9-1.3, n=5)	0.79
GMR_{AUC} (95% CI)				
Placebo group	1 (Reference)	1 (Reference)	1 (Reference)	
RCP group	0.85 (0.69-1.06)	1.06 (0.70-1.61)	1.12 (0.83-1.51)	0.32

Table S13 Geometric means, Geometric Mean Ratio and Geometric Mean Fold Increase for serum IgG antibodies against RBD antigen comparing intranasal RCP to the intranasal placebo stratified by the type of the primary vaccination

	**Razi Cov Pars**	**Sinopharm**	**Spikogen**	**P trend**
GM_{AUC} (95% CI)				
Baseline, placebo group	189807.2 (174038.3-207004.9, n=58)	158768.7 (125440.2-200952.2, n=25)	146613.2 (70544.7-304706.5, n=5)	0.18
Day 14, placebo group	205648.6 (182262.5-232035.3, n=57)	155612.9 (116198.4-208396.9, n=25)	211153.8 (159411.7-279690.4, n=5)	0.15
Baseline , RCP group	180339.4 (162863.2-199690.8, n=60)	171506.1 (135115.7-217697.5, n=24)	189825.8 (122730.8-293600.5, n=4)	0.98
Day 14, RCP group	166095.1 (139228.2-198146.5, n=53)	181627.8 (145675.2-226453.4, n=24)	245464.4 (112308.1-536495.2, n=3)	0.43
$GMFI_{AUC}$ (95% CI)				
Reference (Baseline)	1 (Reference)	1 (Reference)	1 (Reference)	
Day 14, placebo group	1.1 (1.0-1.2, n=54)	1.0 (0.7-1.3, n=24)	1.4 (0.6-3.6, n=5)	0.21
Day 14, RCP group	0.9 (0.7-1.1, n=52)	1.1 (0.8-1.4, n=22)	1.3 (0.9-1.8, n=3)	0.82
GMR_{AUC} (95% CI)				
Placebo group	1 (Reference)	1 (Reference)	1 (Reference)	
RCP group	0.81 (0.66-1.00)	1.17 (0.82-1.67)	1.16 (0.73-1.85)	0.093

Serum ELISA IgA level for SARS-CoV-2 S-antigen

Table S14 Geometric means, Geometric Mean Ratio and Geometric Mean Fold Increase for serum IgA antibodies against S antigen comparing intranasal RCP to the intranasal placebo

	Placebo	Intranasal RCP
GM_{AUC} (95% CI)		
Baseline	85344.2 (78607.3-92658.5, n=91)	94027.1 (86399.3-102328.3, n=95)
Day 14	97939.3 (87858.6-109176.6, n=90)	93169.3 (83327.3-104173.7, n=86)
$GMFI_{AUC}$ (95% CI)		
Baseline	1 (Reference)	1 (Reference)
Day 14	1.1 (1.0-1.3, n=86)	1.0 (0.9-1.1, n=84
GMR_{AUC} (95% CI)		
Baseline	1 (Reference)	1.10 (0.98-1.24)
Day 14	1 (Reference)	0.95 (0.81-1.11)

Figure S2 Scatter plots of individual values and their geometric means for specific IgA antibody levels against S antigen in the serum at the time of vaccination and two weeks after by study groups

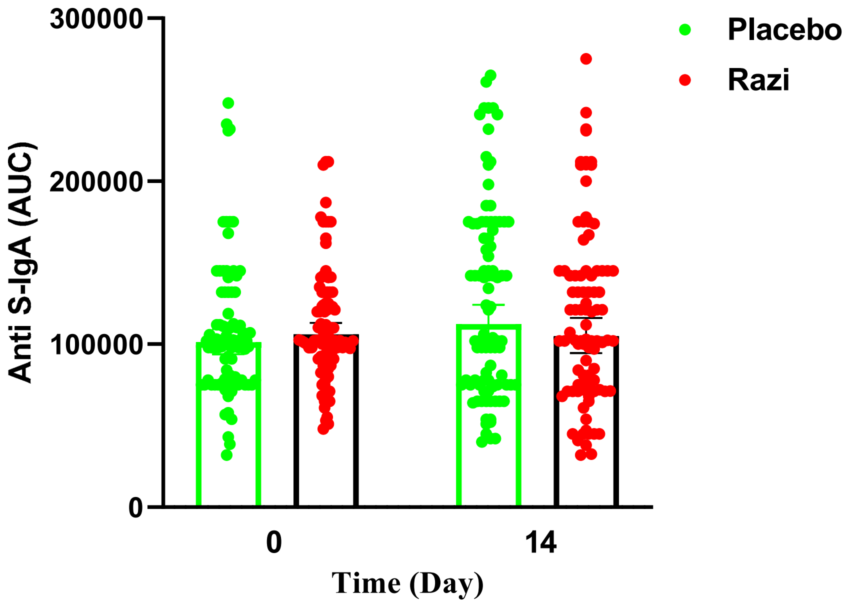

Table S15 Geometric means, Geometric Mean Ratio and Geometric Mean Fold Increase for serum IgA antibodies against S antigen comparing intranasal RCP to the intranasal placebo stratified by the time interval between last vaccination and the intranasal booster dose

	**Tertile 1, n=65 (5 – 9.5 month)**	**Tertile 2, n=64 (9.5 – 12.5 months)**	**Tertile 3, n=64 (12.5 – 18 months)**	**P trend**
GM_{AUC} (95% CI)				
Baseline, placebo group	103384.5 (89246.1-119762.8, n=34)	113212.4 (95394.7-134358.1, n=29)	94238.8 (81794.7-108576.1, n=28)	0.29
Day 14, placebo group	125745.0 (105880.1-149336.9, n=34)	118015.3 (99473.6-140013.2, n=33)	91059.5 (76297.0-108678.5, n=26)	0.02
Baseline , RCP group	104966.9 (93093.8-118354.2, n=30)	106483.3 (87340.9-129821.1, n=29)	113245.9 (102797.2-124756.7, n=36)	0.65
Day 14, RCP group	105019.7 (86818.3-127037.0, n=28)	106683.0 (88982.9-127903.8, n=30)	102903.4 (85860.9-123328.5, n=33)	0.96
$GMFI_{AUC}$ (95% CI)				
Reference (Baseline)	1.00	1.00	1.00	0.15
Day 14, placebo group	1.2 (1.0-1.5, n=33)	1.0 (0.8-1.3, n=29)	1.0 (0.9-1.2, n=26)	0.70
Day 14, RCP group	1.0 (0.9-1.2, n=28)	1.0 (0.8-1.2, n=28)	0.9 (0.8-1.1, n=33)	0.10
GMR_{AUC} (95% CI)				
Placebo group	1 (Reference)	1 (Reference)	1 (Reference)	
RCP group	0.84 (0.65-1.07)	0.90 (0.71-1.15)	1.13 (0.88-1.45)	0.29

Table S16 Geometric means, Geometric Mean Ratio and Geometric Mean Fold Increase for serum IgA antibodies against S antigen comparing intranasal RCP to the intranasal placebo stratified by type of last vaccine received

	**Razi Cov Pars**	**Sinopharm**	**Spikogen**	**P trend**
GM_{AUC} (95% CI)				
Baseline, placebo group	108294.3 (97734.1-119995.4, n=61)	73519.1 (60951.2-88678.6, n=16)	155253.5 (115881.6-208002.4, n=7)	0.031
Day 14, placebo group	122997.6 (108745.3-139117.9, n=61)	90422.0 (72742.8-112397.8, n=17)	120659.8 (78584.9-185261.9, n=8)	0.055
Baseline , RCP group	108284.7 (97469.5-120299.8, n=61)	100821.8 (85926.1-118299.7, n=19)	125060.4 (96387.7-162262.4, n=8)	0.74
Day 14, RCP group	102915.5 (90319.1-117268.7, n=59)	95182.6 (77880.7-116328.2, n=19)	122068.7 (70713.5-210720.2, n=7)	0.34
$GMFI_{AUC}$ (95% CI)				
Reference (Baseline)	1.00	1.00	1.00	
Day 14, placebo group	1.1 (1.0-1.3, n=58)	1.2 (0.9-1.5, n=16)	0.8 (0.6-1.1, n=7)	0.26
Day 14, RCP group	0.9 (0.8-1.1, n=58)	0.9 (0.8-1.1, n=19)	0.9 (0.7-1.4, n=6)	0.82
GMR_{AUC} (95% CI)				
Placebo group	1 (Reference)	1 (Reference)	1 (Reference)	
RCP group	0.84 (0.70-1.00)	1.05 (0.79-1.40)	1.01 (0.55-1.87)	0.28

Table S17 Geometric means, Geometric Mean Ratio and Geometric Mean Fold Increase for serum IgA antibodies against S antigen comparing intranasal RCP to the intranasal placebo stratified by the type of the primary vaccination

	**Razi Cov Pars**	**Sinopharm**	**Spikogen**	**P trend**
GM_{AUC} (95% CI)				
Baseline, placebo group	111885.2 (101813.6-121956.8, n=58)	122697.2 (69224.8-176169.6, n=25)	100450.0 (66475.5-134424.5, n=5)	0.76
Day 14, placebo group	133643.1 (118143.0-149143.1, n=59)	107696.2 (85410.9-129981.4, n=26)	117000.0 (58767.4-175232.6, n=5)	0.15
Baseline , RCP group	120946.4 (92721.8-149171.0, n=60)	112713.7 (97887.3-127540.1, n=25)	119750.0 (45586.8-193913.2, n=4)	0.93
Day 14, RCP group	110956.9 (97250.3-124663.5, n=58)	114212.5 (91513.6-136911.4, n=24)	155000.0 (12693.1322693.1, n=3)	0.37
$GMFI_{AUC}$ (95% CI)				
Reference (Baseline)	1.00	1.00	1.00	
Day 14, placebo group	1.2 (1.1-1.4, n=55)	1.2 (0.8-1.5, n=25)	1.2 (0.5-2.0, n=5)	0.91
Day 14, RCP group	1.1 (0.9-1.2, n=57)	1.0 (0.9-1.2, n=23)	1.3 (0.0-2.5, n=3)	0.32
GMR_{AUC} (95% CI)				
Placebo group	1 (Reference)	1 (Reference)	1 (Reference)	
RCP group	0.82 (0.69-0.98)	1.08 (0.82-1.41)	1.33 (0.64-2.76)	0.062

Serum ELISA IgA level for SARS-CoV-2 RBD-antigen

Table S18 Geometric means, Geometric Mean Ratio and Geometric Mean Fold Increase for serum IgA antibodies against S antigen comparing intranasal RCP to the intranasal placebo stratified by the time interval between last vaccination and the intranasal booster dose

	**Tertile 1, n=65 (5 – 9.5 month)**	**Tertile 2, n=64 (9.5 – 12.5 months)**	**Tertile 3, n=64 (12.5 – 18 months)**	**P trend**
GM_{AUC} (95% CI)				
Baseline, placebo group	86060.1 (73166.1-101226.3, n=34)	88057.8 (78265.0-99075.9, n=29)	81788.1 (70295.1-95160.2, n=28)	0.76
Day 14, placebo group	112286.5 (92530.6-136260.6, n=32)	102251.9 (85526.4-122248.3, n=32)	78496.9 (64986.9-94815.3, n=26)	0.016
Baseline , RCP group	91045.6 (80196.3-103362.7, n=30)	89937.9 (72385.1-111747.0, n=29)	100108.3 (89932.9-111435.1, n=36)	0.68
Day 14, RCP group	90930.8 (73590.8-112356.6, n=25)	95032.1 (78217.8-115461.0, n=29)	93267.9 (76490.8-113724.9, n=32)	0.94
$GMFI_{AUC}$ (95% CI)				
Reference (Baseline)	1.00	1.00	1.00	
Day 14, placebo group	1.3 (1.0-1.6, n=31)	1.1 (1.0-1.4, n=29)	1.0 (0.8-1.2, n=26)	0.13
Day 14, RCP group	1.0 (0.9-1.2, n=25)	1.0 (0.8-1.3, n=27)	0.9 (0.7-1.1, n=32)	0.70
GMR_{AUC} (95% CI)				
Placebo group	1 (Reference)	1 (Reference)	1 (Reference)	
RCP group	0.81 (0.61-1.07)	0.93 (0.72-1.20)	1.19 (0.90-1.56)	0.053

Table S19 Geometric means, Geometric Mean Ratio and Geometric Mean Fold Increase for serum IgA antibodies against S antigen comparing intranasal RCP to the intranasal placebo stratified by type of last vaccine received

	**Razi Cov Pars**	**Sinopharm**	**Spikogen**	**P trend**
GM_{AUC} (95% CI)				
Baseline, placebo group	88527.8 (80867.7-96913.5, n=61)	61009.2 (49543.2-75128.7, n=16)	130797.6 (96457.7-177362.9, n=7)	<0.001
Day 14, placebo group	107442.1 (94254.2-122475.2, n=59)	77833.6 (59543.3-101742.2, n=16)	106275.8 (67538.9-167230.1, n=8)	0.082
Baseline , RCP group	93096.0 (82864.7-104590.5, n=61)	88518.4 (74651.3-104961.3, n=19)	110957.7 (83427.7-147572.2, n=8)	0.78
Day 14, RCP group	90685.8 (78710.4-104483.2, n=55)	81348.8 (63877.9-103598.0, n=19)	138810.9 (98448.0-195722.4, n=6)	0.65
$GMFI_{AUC}$ (95% CI)				
Reference (Baseline)	1.00	1.00	1.00	
Day 14, placebo group	1.2 (1.1-1.4, n=57)	1.2 (0.9-1.7, n=15)	0.8 (0.6-1.2, n=7)	0.27
Day 14, RCP group	1.0 (0.8-1.1, n=54)	0.9 (0.8-1.1, n=19)	1.1 (0.8-1.6, n=5)	0.65
GMR_{AUC} (95% CI)				
Placebo group	1 (Reference)	1 (Reference)	1 (Reference)	
RCP group	0.84 (0.70-1.02)	1.05 (0.74-1.48)	1.31 (0.76-2.26)	0.14

Table S20 Geometric means, Geometric Mean Ratio and Geometric Mean Fold Increase for serum IgA antibodies against S antigen comparing intranasal RCP to the intranasal placebo stratified by the type of the primary vaccination

	**Razi Cov Pars**	**Sinopharm**	**Spikogen**	**P trend**
GM_{AUC} (95% CI)				
Baseline, placebo group	90068.0 (82226.2-98657.7, n=58)	74655.8 (61521.8-90593.7, n=25)	81933.5 (57932.5-115877.8, n=5)	0.30
Day 14, placebo group	104577.4 (91864.8-119049.1, n=57)	84063.1 (67081.6-105343.4, n=25)	95414.4 (56318.4-161650.4, n=5)	0.25
Baseline , RCP group	91278.1 (81192.5-102616.6, n=60)	94425.6 (81431.2-109493.6, n=25)	96914.6 (53636.7-175112.4, n=4)	0.95
Day 14, RCP group	87773.4 (76120.5-101210.2, n=53)	90308.6 (72208.3-112946.2, n=24)	132147.8 (48001.1-363805.6, n=3)	0.58
$GMFI_{AUC}$ (95% CI)				
Reference (Baseline)	1.00	1.00	1.00	
Day 14, placebo group	1.2 (1.0-1.3, n=54)	1.1 (0.9-1.4, n=24)	1.2 (0.5-2.5, n=5)	0.98
Day 14, RCP group	0.9 (0.8-1.1, n=52)	1.0 (0.8-1.1, n=23)	1.3 (0.5-3.3, n=3)	0.58
GMR_{AUC} (95% CI)				
Placebo group	1 (Reference)	1 (Reference)	1 (Reference)	
RCP group	0.84 (0.69-1.01)	1.07 (0.79-1.46)	1.38 (0.66-2.93)	0.11

Saliva ELISA IgA level for SARS-CoV-2 RBD-antigen

Table S21 Geometric means, Geometric Mean Ratio and Geometric Mean Fold Increase for saliva IgA antibodies against RBD antigen comparing intranasal RCP to the intranasal placebo

	Placebo	Intranasal RCP
GM_{AUC} (95% CI)		
Baseline	31911.1 (31016.2-32831.9, n=88)	31339.5 (30648.5-32046.1, n=93)
Day 14	32618.6 (31843.7-33412.4, n=94)	33388.7 (32285.1-34530.0, n=92)
$GMFI_{AUC}$ (95% CI)		
Baseline	1 (Reference)	1 (Reference)
Day 14	1.0 (1.0-1.1, n=86)	1.1 (1.0-1.1, n=88)
GMR_{AUC} (95% CI)		
Baseline	1 (Reference)	0.98 (0.95-1.02)
Day 14	1 (Reference)	1.02 (0.98-1.07)

Figure S3 Scatter plots of individual values and their geometric means for specific IgA antibody levels against RBD antigen in the saliva at the time of vaccination and two weeks after by study groups

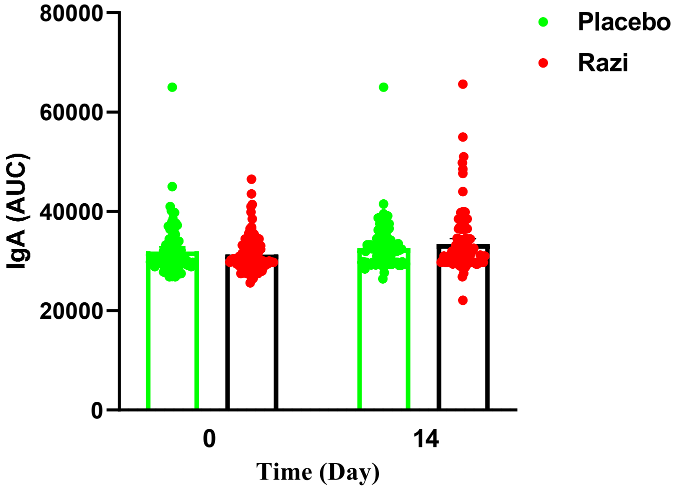

Nasal mucosal ELISA IgA level for SARS-CoV-2 RBD-antigen

Table S22 Geometric means, Geometric Mean Ratio and Geometric Mean Fold Increase for nasal mucosal IgA antibodies against RBD antigen comparing intranasal RCP to the intranasal placebo stratified by the time interval between last vaccination and the intranasal booster dose

	**Tertile 1, n=65 (5 – 9.5 month)**	**Tertile 2, n=64 (9.5 – 12.5 months)**	**Tertile 3, n=64 (12.5 – 18 months)**	**P trend**
GM_{AUC} (95% CI)				
Baseline, placebo group	31522.6 (25283.0-39301.9, n=33)	38740.1 (31447.7-47723.6, n=30)	34045.2 (26640.3-43508.3, n=28)	0.54
Day 14, placebo group	38816.3 (30987.7-48622.6, n=34)	40757.2 (32269.7-51477.0, n=32)	31323.5 (24223.5-40504.6, n=26)	0.34
Baseline , RCP group	34898.8 (28565.8-42635.9, n=29)	32618.1 (26499.7-40149.2, n=29)	44666.8 (37035.9-53870.0, n=36)	0.04
Day 14, RCP group	50107.9 (36617.4-68568.7, n=27)	43265.7 (34508.1-54245.9, n=30)	47449.1 (36646.1-61436.8, n=33)	0.36
$GMFI_{AUC}$ (95% CI)				
Reference (Baseline)	1.00	1.00	1.00	
Day 14, placebo group	1.2 (1.0-1.5, n=32)	1.0 (0.8-1.3, n=29)	1.0 (0.8-1.3, n=26)	0.53
Day 14, RCP group	1.5 (1.1-2.0, n=26)	1.3 (1.1-1.7, n=28)	1.0 (0.9-1.2, n=33)	0.07
GMR_{AUC} (95% CI)				
Placebo group	1 (Reference)	1 (Reference)	1 (Reference)	
RCP group	1.29 (0.89-1.87)	1.06 (0.77-1.46)	1.51 (1.05-2.18)	0.84

Table S23 Geometric means, Geometric Mean Ratio and Geometric Mean Fold Increase for nasal mucosal IgA antibodies against RBD antigen comparing intranasal RCP to the intranasal placebo stratified by type of last vaccine received

	**Razi Cov Pars**	**Sinopharm**	**Spikogen**	**P trend**
GM_{AUC} (95% CI)				
Baseline, placebo group	32633.7 (28063.9-37947.6, n=61)	38600.4 (28411.4-52443.4, n=16)	40615.8 (18081.0-91236.3, n=7)	0.23
Day 14, placebo group	35319.1 (29942.8-41660.7, n=60)	42814.3 (29327.8-62502.7, n=17)	44920.9 (31226.7-64620.6, n=8)	0.35
Baseline , RCP group	35589.6 (30946.4-40929.5, n=60)	43140.5 (31745.2-58626.2, n=18)	36015.7 (22538.3-57552.3, n=9)	0.35
Day 14, RCP group	43254.2 (35328.3-52958.2, n=58)	53336.9 (39920.0-71263.1, n=19)	65134.3 (39534.0-107312.1, n=7)	0.72
$GMFI_{AUC}$ (95% CI)				
Reference (Baseline)	1.00	1.00	1.00	
Day 14, placebo group	1.1 (0.9-1.3, n=57)	1.1 (0.8-1.5, n=16)	1.0 (0.5-2.1, n=7)	0.96
Day 14, RCP group	1.2 (1.0-1.5, n=56)	1.2 (0.9-1.6, n=18)	1.8 (1.1-3.1, n=7)	0.60
GMR_{AUC} (95% CI)				
Placebo group	1 (Reference)	1 (Reference)	1 (Reference)	
RCP group	1.22 (0.95-1.58)	1.25 (0.79-1.96)	1.45 (0.84-2.50	0.68

Table S24 Geometric means, Geometric Mean Ratio and Geometric Mean Fold Increase for nasal mucosal IgA antibodies against RBD antigen comparing intranasal RCP to the intranasal placebo stratified by the type of the primary vaccination

	**Razi Cov Pars**	**Sinopharm**	**Spikogen**	**P trend**
GM_{AUC} (95% CI)				
Baseline, placebo group	30761.6 (26202.8-36113.6, n=58)	45344.5 (36390.3-56502.1, n=25)	45538.9 (25849.8-80224.6, n=5)	0.06
Day 14, placebo group	34193.1 (29277.4-39934.1, n=59)	42184.2 (31250.0-56944.4, n=25)	52672.6 (22435.1-123663.6, n=5)	0.06
Baseline , RCP group	35103.6 (30558.1-40325.3, n=59)	42815.7 (33039.9-55484.0, n=25)	33180.3 (20398.4-53971.4, n=4)	0.15
Day 14, RCP group	42038.3 (34425.6-51334.4, n=58)	59097.6 (45501.4-76756.5, n=23)	40074.8 (15502.0-103598.7, n=3)	0.48
$GMFI_{AUC}$ (95% CI)				
Reference (Baseline)	1.00	1.00	1.00	
Day 14, placebo group	1.1 (0.9-1.3, n=55)	0.9 (0.7-1.2, n=24)	1.2 (0.6-2.4, n=5)	0.38
Day 14, RCP group	1.2 (1.0-1.5, n=56)	1.3 (1.0-1.7, n=22)	1.1 (0.3-4.4, n=3)	0.90
GMR_{AUC} (95% CI)				
Placebo group	1 (Reference)	1 (Reference)	1 (Reference)	
RCP group	1.23 (0.96-1.58)	1.40 (0.95-2.07)	0.76 (0.26-2.24)	0.92

Nasal mucosal ELISA IgA level for Omicron variant of SARS-CoV-2 S-antigen

Table S25 Geometric means, Geometric Mean Ratio and Geometric Mean Fold Increase for nasal mucosal IgA antibodies against RBD antigen from Omicron variant of SARS-CoV-2 comparing intranasal RCP to the intranasal placebo

	Placebo	Intranasal RCP
GM_{AUC} (95% CI)		
Baseline	25582.7 (21740.1-30104.5, n=91)	26141.2 (23220.4-29429.3, n=93)
Day 14	30218.6 (25837.0-35343.3, n=92)	51826.2 (40897.5-65675.2, n=90)
$GMFI_{AUC}$ (95% CI)		
Baseline	1 (Reference)	1 (Reference)
Day 14	1.2 (1.0-1.4, n=87)	2.0 (1.6-2.6, n=86)
GMR_{AUC} (95% CI)		
Baseline	1 (Reference)	1.02 (0.84-1.25)
Day 14	1 (Reference)	1.72 (1.30-2.27)

Figure S4 Scatter plots of individual values and their geometric means for specific IgA antibody levels against RBD antigen from Omicron variant of SARS-CoV-2 in the nasal mucosa at the time of vaccination and two weeks after by study groups

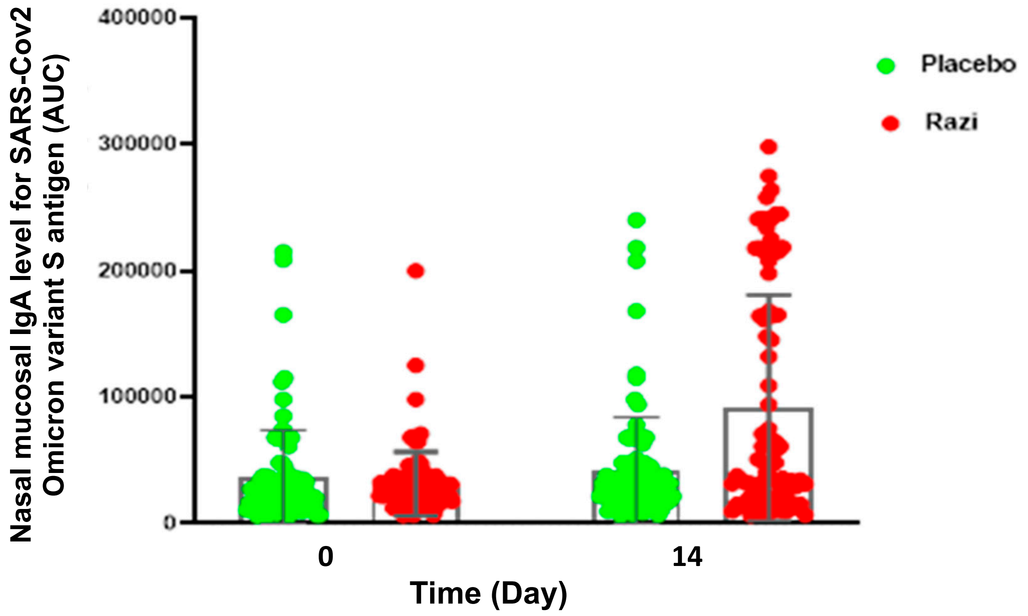

Nasal mucosal ELISA IgA level for Wuhan variant of SARS-CoV-2 S-antigen

Table S26 Geometric means, Geometric Mean Ratio and Geometric Mean Fold Increase for nasal mucosal IgA antibodies against RBD antigen from Wuhan variant of SARS-CoV-2 comparing intranasal RCP to the intranasal placebo

	Placebo	Intranasal RCP
GM_{AUC} (95% CI)		
Baseline	29369.0 (24881.8-34665.5, n=91)	28434.9 (25116.2-32192.1, n=93)
Day 14	36403.5 (31115.3-42590.6, n=92)	61669.4 (49236.2-77242.3, n=90)
$GMFI_{AUC}$ (95% CI)		
Baseline	1 (Reference)	1 (Reference)
Day 14	1.2 (1.0-1.5, n=87)	2.2 (1.8-2.7, n=86)
GMR_{AUC} (95% CI)		
Baseline	1 (Reference)	0.97 (0.79-1.19)
Day 14	1 (Reference)	1.69 (1.29-2.22)

Figure S5 Scatter plots of individual values and their geometric means for specific IgA antibody levels against RBD antigen from Wuhan variant of SARS-CoV-2 in the nasal mucosa at the time of vaccination and two weeks after by study groups

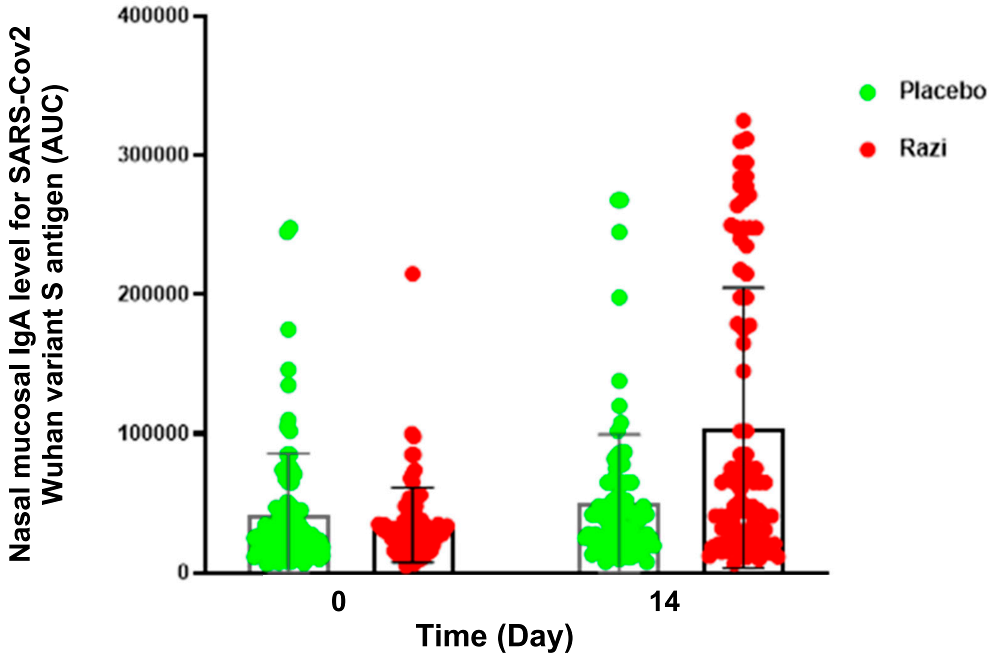

Results for a subgroup of 100 participants from Phase III clinical trial study

Saliva and serum IgA level for SARS-CoV-2 S antigen

Figure S6 Scatter plots of individual values and their geometric means of anti-RBD specific IgA antibody in saliva of a subpopulation of phase III study participants in RAZI and Sinopharm groups in response to IN RCP or placebo, respectively, at the time of IN booster vaccination and two weeks later

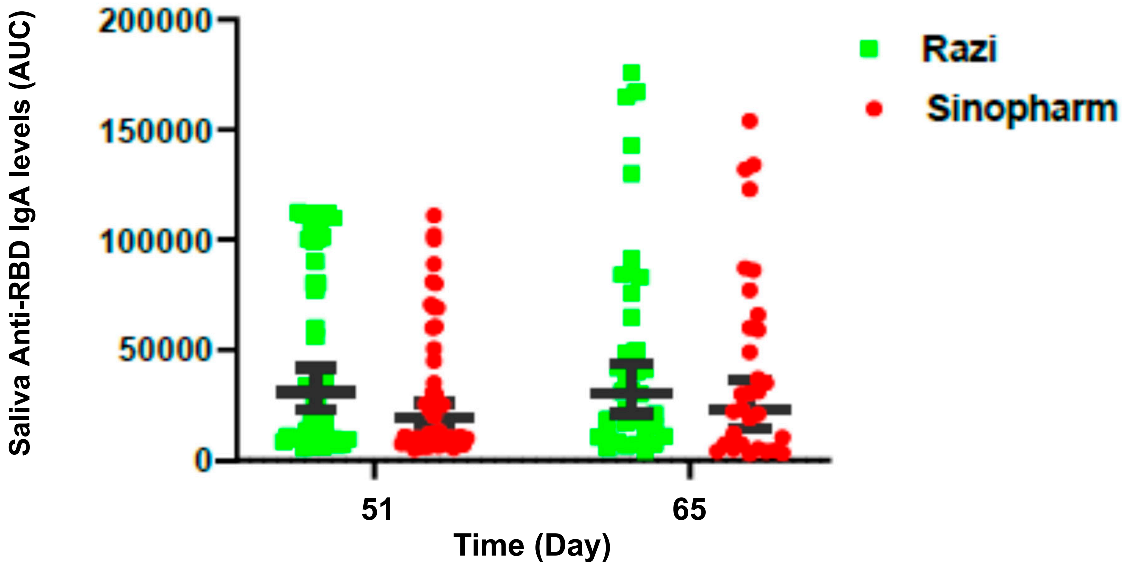

Figure S7 Scatter plots of individual values and their geometric means of anti-RBD specific IgA antibody in serum of a subpopulation of phase III study participants in RAZI and Sinopharm groups in response to IN RCP or placebo, respectively, at the time of IN booster vaccination and two weeks later

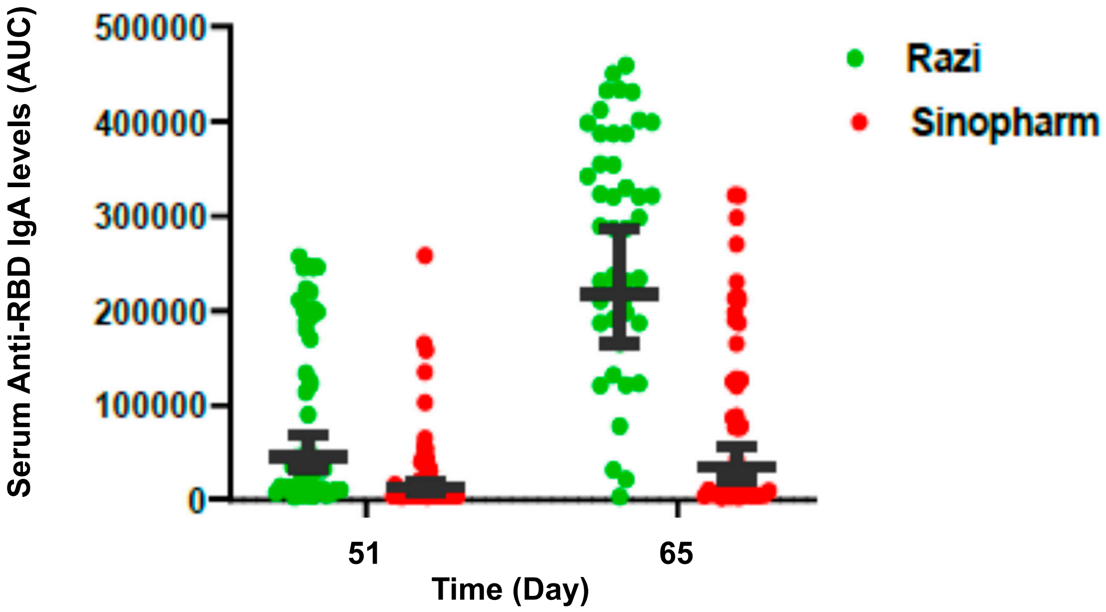

Supplement: Supplementary file 1 [file vaccines-12-01255-s001.zip › vaccines-3187491-supplementary.pdf]
